# Isolation and characterization of novel microorganisms producing natural compounds of possible industrial interest: an integrated genomic and metabolomic approach

**DOI:** 10.3389/fmicb.2026.1872113

**Published:** 2026-07-08

**Authors:** Andrea Nicolò Dell’Acqua, Giorgia Palladino, Simone Rampelli, Daniela Leuzzi, Daniel Scicchitano, Rosa Doménech, Ana Mencher, Thais Delgado, Martín Perales, Cinzia Corinaldesi, Arianna Mancuso, Stefano Goffredo, Emanuele Porru, Jessica Fiori, Marco Candela

**Affiliations:** 1 Unit of Microbiome Science and Biotechnology, Department of Pharmacy and Biotechnology, University of Bologna, Bologna, Italy; 2 Fano Marine Center, The Inter-Institute Center for Research on Marine Biodiversity, Resources and Biotechnologies, Fano, Pesaro Urbino, Italy; 3 Instituto Tecnológico del Embalaje, Transporte y Logística (ITENE), Paterna, Spain; 4 Department of Sciences and Engineering of Materials, Environment and Urbanistics, Polytechnic University of Marche, Ancona, Italy; 5 Marine Science Group, Department of Biological, Geological and Environmental Sciences, University of Bologna, Bologna, Italy; 6 Occupational Medicine Unit, Department of Medical and Surgical Sciences, University of Bologna, Bologna, Italy; 7 Department of Chemistry “G. Ciamician”, University of Bologna, Bologna, Italy

**Keywords:** bioactive natural compounds, extreme environments, microorganisms isolation, omics-driven analysis, pigmented microorganisms, sustainable production

## Abstract

**Background:**

This study investigates the discovery of bioactive natural compound (BNC)-producing microorganisms from diverse, marine and terrestrial extreme environments using an integrated genomic and metabolomic approach. BNCs, including pigments, antimicrobials, and antioxidants, play pivotal roles in microbial adaptation and constitute valuable resources for applications spanning pharmaceuticals to sustainable materials. To enhance the likelihood of isolating BMC producers, a pigmentation-guided strategy was applied across three ecologically distinct and extreme environments: alpine glacier snow, volcanic marine sediments, and marine holobionts exposed to natural CO_2_ emissions.

**Results:**

This approach yielded 51 pigmented microbial isolates, encompassing both bacteria and fungi across multiple phyla. From this collection, seven representative strains exhibiting high growth rate and consistent and intense pigmentation were selected for in-depth characterization. Whole-genome sequencing and biosynthetic gene cluster (BGC) analysis revealed extensive biosynthetic potential, with 61 BGCs identified, including pathways associated with terpenes, polyketide synthases (PKS), and non-ribosomal peptide synthetases (NRPS). Complementary metabolomic profiling via UPLC-QTOF-MS confirmed the production of a broad spectrum of BNC, with 58 natural products being detected, including carotenoids, antimicrobial compounds, biosurfactants, and polyketide-derived pigments. Functional assays further demonstrated the ability of these strains to metabolize amylaceous substrates and plant-derived carbohydrates, highlighting their suitability for sustainable biotechnological processes based on renewable feedstocks. Notably, two isolates affiliated with *Alkalihalobacillus algicola* and *Cytobacillus oceanosediminis* exhibited genomic divergence below species-level thresholds, suggesting the presence of previously uncharacterized lineages.

**Conclusion:**

Overall, this work underscores the effectiveness of coupling pigmentation-based screening with omics-driven analyses to uncover microorganisms as candidate cell factory for BNC production. The identified strains represent promising candidates for sustainable biotechnological applications, including the production of natural pigments, antimicrobials, and bio-based materials, thereby contributing to the advancement of circular and eco-friendly industrial systems.

## 1 Background

Bioactive natural compounds (BNCs) refer to molecules produced by plants, fungi, microorganisms, or other natural sources that demonstrate a biologically relevant activity (Jin et al., 2016; Denaro et al., 2020). These compounds, generated by means of secondary metabolism, are not directly involved in energy production and cellular growth, lacking a role in the internal economy of the producing organisms (Williams et al., 1989; Williams and Maplestone, 1992). Conversely, BNCs are crucial for the survival in the natural environment, as they represent a strategic adaptive and defensive system against varying biotic or abiotic stressors, ultimately increasing fitness (Firn and Jones, 2000; Tyc et al., 2017). As such, BNCs are not part of a single molecular class, but of a range of widely chemically different classes, showing properties such as antimicrobial, antiviral, antifouling, cytotoxic, antioxidant, UV-protective compounds or siderophores (Atanasov et al., 2021). Historically, research on BNCs focused primarily on health-related and clinical applications, where they constituted over 70% of antibacterial drugs available on the market as of 2014 (Khan et al., 2014; Newman and Cragg, 2016). More recently, the use of BNCs is expanding to different sectors, other than pharmaceuticals, for example with applications in the cosmetic sector, where BNCs as carotenoids and flavonoids can be used to improve the final appearance of the product or provide a skin lightening effect (Dasgupta Mandal and Majumdar, 2023; Sharma et al., 2024). BNCs can also be used as food additives, where they are used as natural dyes or antioxidants compounds for the formulation of food supplements and nutraceuticals (Al-Obaidi et al., 2021; Barsby et al., 2021; López-Rodríguez et al., 2022). Secondary metabolites, such as carotenoids, can also be employed for the production of intelligent packages, where their addition in protective films can reduce oxidation and increase the preservation of food, or where they could act as sensors, indicating when a significative change in the quality of food has occurred (Leccese et al., 2023; Roy et al., 2023). Lastly, pigmented BNCs are being investigated as potential natural substitutes for artificial pigments and dyes in the textile industry. Artificial pigments currently represent a major source of environmental and water pollution (De Oliveira et al., 2013). With their excellent color fastness, properties and ability to meet industry standards, natural pigments represent promising candidates as sustainable alternatives (Ackacha et al., 2003; Duval et al., 2016).

From the genomic perspective, microbial BNCs are produced by biosynthetic gene clusters (BGCs), complex assemblies of clustered biosynthetic and regulatory genes that encode for the complete machinery for BNC production. BGCs are usually organized around a core biosynthetic gene, encoding for a key multimodule protein responsible for the production of BNC molecular scaffold. Accessory genes encoding tailoring enzymes transporters, resistance genes, and regulatory elements are typically associated with the core biosynthetic genes (Cimermancic et al., 2014). Depending on the specific BGCs class, the core gene may encode non-ribosomal peptide synthases (NRPS) or polyketide synthases (PKS). Other known BCN classes include terpenes and ribosomally synthesized and post-translationally modified peptide (RiPPs) (Sedkova et al., 2005; Oldfield and Lin, 2012; Zdouc et al., 2023; Li H. et al., 2024).

In our work, we explored diverse microbiomes from marine and terrestrial extreme environments to identify novel BNC producers, given that BNCs are frequently produced in response to specific environmental stressors. The selected microbiomes included: (i) microbial communities from glacier ecosystems, characterized by low temperatures, high UV radiation, and oligotrophic conditions; (ii) microbiomes from volcanic coastal marine sediments influenced by shallow hydrothermal CO_2_ rich venting, with locally sulfidic conditions near active seeps and (iii) microbiomes associated with marine holobionts, such as corals, that rely on their associated microbiomes for acclimatization and functional resilience under chronic acidification stress (Palladino et al., 2022). These included the corals B*alanophyllia europaea* and *Caryophyllia inornata*, inhabiting naturally acidified waters near CO_2_ vents and vent caves, as well as the seagrass *Posidonia oceanica*, living approximately 30 m from the vent. To enrich for secondary-metabolite producers, we used a pigmentation-guided isolation strategy, where colony color served as a proxy for active secondary metabolism (Pailliè-Jiménez et al., 2020; Mussagy et al., 2022; Dasgupta Mandal and Majumdar, 2023; Sharma et al., 2024). Fermentable pigmented isolates were selected for in-depth investigation as microbial cell factories through an integrated approach combining whole-genome sequencing, BGCs mining, and Ultra-Performance Liquid Chromatography–Mass Spectrometry (UPLC–MS) profiling of secondary metabolites. This strategy enabled the discovery of novel microorganisms that might be implemented as inherently robust platforms for the sustainable production of pigments and bioactive compounds.

## 2 Materials and methods

### 2.1 Sites description and samples collection

For the isolation of novel bioactive natural compounds (BNCs) producing microbial strains, three different extreme environments were selected. The Presena Glacier, located in Trentino Alto Adige, Italy, reaches an altitude of 3,000 m a.s.l. and is characterized by freezing temperatures, high UV radiation exposure, freeze-thaw cycles in the upper ice layers, and limited nutrients availability (Rekadwad et al., 2024; Crosta et al., 2025). Snow at the Presena Glacier was collected in “Passo Presena” inside 2 L sterile bottles using sterilized tools. Samples were transported frozen to the laboratory and kept at -20 °C until further processing. Coastal marine sediments at the volcanic island of Vulcano were influenced by shallow hydrothermal CO_2_ rich venting with sulfidic conditions, located off the coasts of Sicily, Italy. The presence of volcanic activity in the form of sulphureous fumaroles confers the surrounding water with a unique composition of dissolved minerals and more acidic pH (Rusch et al., 2005; Kerfahi et al., 2014). Samples were retrieved using three 50 mL sterile falcon tubes and transported at 4 °C to the lab, where they were immediately processed. Further, potential BNCs producing microorganisms were retrieved from individuals of the corals *Balanophyllia europaea* and *Caryophyllia inornata*, and from leaves of the seagrass *Poseidonia oceanica*, located at different underwater sites in Panarea island, Sicily, Italy. Five *B. europaea* individuals and 10 *P. oceanica* individuals were collected at an underwater CO_2_ crater at 10 m depth, releasing persistent gaseous emissions (98–99% CO_2_ without instrumentally detectable taxic compounds) as described by Palladino et al. (2022). Sampling sites along this gradient match mean pH values projected for 2,100 under different IPCC scenarios (Chen et al., 2021). Five individuals of *C. inornata* were collected at a submerged cave located at a depth of 14 m off Basiluzzo Island. The site is characterized by hydrothermal emissions sweeping from the seabed in proximity and within the cave, as described by Cassarino et al. (2025). Coral and seagrass samples were transported alive to the laboratory inside 1 L seawater tanks 2 days after the sampling. Seawater was changed daily using fresh backup seawater collected from the same sampling site, and samples were processed immediately upon arrival.

### 2.2 Isolation of pigmented microorganisms

For the isolation of pigmented microorganisms, samples were processed according to the matrix type. Snow collected at the Presena Glacier was melted at 4 °C and centrifuged at 9,000 g for 5 min. The resulting pellet was resuspended in 10 mL of sterile water. 100 μL of resuspended sample were plated on TSA and Vogel’s medium N and incubated at 18 °C (Mortazavi et al., 2008; Mortazavi et al., 2014). For isolation from marine sediments, 10-fold serial dilutions from 10^–1^ to 10^–5^ were prepared in artificial seawater (ASW). Starting from 1 g of resuspended sediments, 100 μL of each dilution were plated on TSA, MA, and Vogel’s medium N agar (Romanenko et al., 2023) and incubated at 22 °C (Radovanović et al., 2018; Romanenko et al., 2023). For both *B. europaea* and *C. inornata*, individual polyps were separated and cleaned of algae and debris using sterile tweezers and scalpels. Surface sterilization was performed by rinsing in sterile ASW, immersion in 70% ethanol for 3 s, and a final rinse in new sterile ASW. Following surface sterilization, each polyp was ground using a mortar and pestle to obtain a homogeneous paste. The coral paste was then serially diluted using ASW (Kjer et al., 2010). The same protocol was applied to *Posidonia oceanica* leafes with 1 s immersion in 70% ethanol. Samples of ground up corals and seagrass were serially diluted in ASW from 10^–1^ to 10^–5^. 100 μL of each dilution were plated on TSA, MA, and Vogel’s medium N plates and incubated at 25 °C (Kjer et al., 2010; Susilowati et al., 2021; Wang et al., 2022). Isolated pigmented microbial colonies of interest were picked, streaked onto new plates of the same medium, and incubated under the same conditions as isolation. From pure culture plates, flask liquid cultures were prepared using 30 mL of non-agarized medium (same type and temperature as isolation), with agitation at 100 rpm.

### 2.3 Taxonomical identification and whole genome sequencing

Genomic DNA was extracted from 3 mL of liquid culture for each strain after pelleting at 9,000 g × 5 min using the DNeasy Blood and Tissue Kit (QIAGEN) following manufacturer’s instructions and quantified using a NanoDrop ND1000 spectrophotometer (NanoDrop Technologies, Wilmington, DE, United States). DNA was then diluted in PCR-grade water to a final concentration of 5 ng/μL prior to PCR amplification. The bacterial 16S rRNA gene and fungal ITS regions were targeted for PCR amplicons using universal primers Bact-27F (5’-AGAGTTTGATCMTGGCTCAG-3’)/Bact-1492R (5′- TACGGCTACCTTGTTACGACTT-3′) and ITS-1F (5′-CTTGGTCATTTAGAGGAAGTAA-3′)/ITS-4R (5′-TCCTCCGCTTATTGATATGC-3′) for bacteria and fungi, respectively. Amplicons were purified and sequenced on the Sanger platform. Raw sequencing data were visualized and quality-trimmed using Teal (Rausch et al., 2020). Validated reads were queried against the NCBI nucleotide database via BLASTn (Altschul et al., 1990). Taxonomic assignments were based on hits with > 95% query coverage and percent identity, with E-values < 10^–5^. For 16S rRNA and ITS phylogenetic analyses, the 16S rRNA gene and ITS sequences obtained from the isolates were compared against reference sequences retrieved from LPSN and UNITE databases for bacteria and fungi. To improve tree readability while preserving taxonomic context, for each isolate the closest available reference sequence was selected preferentially at the species level and, when unavailable, at the genus level. Sequences were aligned using MAFFT v7 (Katoh and Standley, 2013) with automatic parameter selection. The resulting alignments were refined using TrimAl v1.4 (Capella-Gutiérrez et al., 2009) with the automated1 option to remove poorly aligned regions. Maximum-likelihood phylogenetic trees were inferred using IQ-TREE v2 (Minh et al., 2020). The best-fit nucleotide substitution model was selected using ModelFinder, and node support was assessed with 1,000 ultrafast bootstrap replicates and 1,000 SH-aLRT replicates. The resulting trees were used to evaluate the phylogenetic relationships between the isolates and their closest reference strains. Seven pigmented microbial isolates were selected for downstream analyses based on their intense and stable pigmentation and robust growth in liquid culture (Supplementary Figure 1): *Rhodosporidiobolus colostri*, *Cytobacillus oceanosediminis*, *Micrococcus yunnanensis*, and *Alkalihalobacillus algicola*, *Paracoccus marcusii*, *Planococcus glaciei*, and *Cladosporium* sp. Each selected strain was processed for shotgun genomic sequencing. DNA libraries were prepared using the QIAseq FX DNA library kit (QIAGEN) according to manufacturer’s instructions. Briefly, 100 ng of input DNA was enzymatically fragmented to 450 bp size, end-repaired, and A-tailed using FX enzyme mix with the following thermal cycle: 4°C for 1 min, 32°C for 8 min, and 65°C for 30 min. Adapter ligation was performed by incubating DNA samples at 20°C for 15 min with DNA ligase and Illumina adapter barcodes. A first purification step was performed usign Agencourt AMPure XP magnetic beads (Backman Coulter), followed by library amplification with limited-cycle PCR according to manufacturer’s instruction, and a further purification step. Samples were then pooled at 4 nM to obtain the final library, and sequencing was performed on an Illumina NextSeq platform using a 2 × 150-bp paired-end protocol, following the manufacturer’s instructions (Illumina, San Diego, CA, United States).

### 2.4 Genome assembly, phylogenetic reconstruction, BGCs mining and Carbohydrate active enzymes (CAZymes) profiling

Raw paired-end reads Illumina reads were quality-filtered using fastp (Chen et al., 2018; v.0.20.1) with parameters –detect_adapter_for_pe –cut_tail -c -D for adapter trimming, low-quality base removal, read deduplication, and base correction. High quality reads were assembled *de novo* with Unicycler (v.0.5.0; Wick et al., 2017), to optimize contig bridging and assembly completeness. Assemblies were evaluated with QUAST (Gurevich et al., 2013; v.5.0.2) for quality metrics (N50, L50, contigs longer than 1,000 bp). Taxonomic classification was performed using GTDB-Tk (v.2.1.1—Genome Taxonomy Database, release 214) (Chaumeil et al., 2020) to confirm Sanger’s results for bacteria. Reference genomes for *Alkalihalobacillus*, *Cytobacillus*, *Micrococcus*, *Paracoccus*, and *Planococcus* genera were downloaded from NCBI RefSeq (last access March 2026) and dereplicated against our assemblies using dRep (Olm et al., 2017; v.3.5.0) with the following parameters -comp 90 -con 5 -sa 0.99 -pa 0.90. Fungal genomes (*Cladosporium* and *Rhodosporidiobolus*) were sourced from NCBI GenBank, quality-checked with BUSCO (Manni et al., 2021; Zdobnov et al., 2021; v.5.0.0; fungi_odb10) and dereplicated against our assemblies using dRep (-sa 0.95, –ignoreGenomeQuality). Secondary metabolite BGCs were predicted using antiSMASH (v.7.1.0) (Blin et al., 2023) with parameters –cb-knownclusters –cb-subclusters –rre –cc-mibig –genefinding-tool prodigal –fullhmmer. Average nucleotide identity (ANI) based distance matrices informed preliminary clustering and dendrogram construction. Clustering was performed using the unweighted pair group method with arithmetic mean and resulting dendrograms were visualized and annotated. The topology of the dendrograms was used to assess the relationships between the isolates and their closest reference genomes, according to the ANI distance values. For CAZymes profiling, Open Reading Frames (ORFs) were predicted from bacterial metagenomic assemblies using Prodigal (v2.6.3; Hyatt et al., 2010), whereas Funannotate (v1.8.17; Palmer and Stajich, 2020) for fungal assemblies. ORFs were subsequently annotated against the dbCAN database using the rundbCAN tool (v5.2.9; Zheng et al., 2023) to identify CAZymes families. For *Cytobacillus oceanisediminis* and *Micrococcus yunnanensis*, for which > 20 sequenced genomes were available in public databases, an in-deep comparative genomic assessment was carried out. To this aim, the genomes assemblies generated in this study, together with publicly available genomes retrieved from GenBank, were structurally and functionally annotated using Prokka v1.14 (Seemann, 2014). The resulting GFF annotation files were used as input for Panaroo v1.5 with default parameters (Tonkin-Hill et al., 2020) to reconstruct the pangenome and generate a binary gene presence/absence matrix. Genome similarity was evaluated using the Jaccard distance calculated from the gene presence/absence matrix. Relationships among genomes were explored through Principal Coordinates Analysis (PCoA) based on the Jaccard distance matrix. In addition, Neighbor-Joining (NJ) and Unweighted Pair Group Method with Arithmetic Mean (UPGMA) clustering trees were generated from the same distance matrix to visualize the overall genomic and genetic relatedness among isolates and reference strains. All graphical representations were generated in R using the packages ggplot2 (Wickham, 2016), ggtree (Yu et al., 2017), ape (Paradis and Schliep, 2019), and vegan (Oksanen et al., 2013).

### 2.5 Carbon source utilization and growth kinetics

The ability of each strain to utilize different carbon sources under aerobic conditions was evaluated using API 50 CH strips (bioMérieux, Marcy-l’Étoile, France) following the manufacturer’s instructions. Briefly, a single colony from each strain was suspended in the provided CBH/E medium and inoculated into all cupules of the strips. Assembled strips were incubated at 25 °C and monitored after 24 and 48 h with color changes recorded for substrate utilization. Growth kinetics were determined for all strains except *Cladosporium* sp. in liquid culture. Strains were grown in tryptone soya broth (TSB) (*Micrococcus yunnannensis* and *Cytobacillus oceanosediminis* at 30°C, *Rhodosporidiobolus colostri* at 20°C) or marine broth (MB) (*Alkalihalo algicola* at 30°C, *Planococcus glacei* and *Paracoccus marcusii* at 25°C). All cultures were incubated in baffled flasks for 34 h, 100 RPM, in a volume of 30 mL of liquid media, with optical density measured at 600 nm (OD_600_) every 2 h using a spectrophotomer. Growth curves were fitted in R (v4.5.0) using the “growthcurver” package (v.0.3.1; Sprouffske and Wagner, 2016). For biomass production at lab scale, *Cladosporium* was cultured with 3 mm glass beads in the flask to further release of mycelia fragments, thus, improving it’s dispersion in the culture broth. All flasks were cultured with passive aeration, by providing the culture flasks with cotton caps, hence allowing the oxygen diffusion.

### 2.6 Biomass preparation and analytical profiling of secondary metabolites

Batch fermentations (96 h) were performed for each strain under the same conditions used for growth kinetics. Fermentations were further scaled up (1 L) under laboratory-scale conditions to assess pigment recovery and analytical profiling in volumes relevant for downstream processing. Post-fermentation, cells were harvested by centrifugation at 9,000 g for 5 min and wet biomass weight determined. Biomass was resuspended (1 g in 3 mL distilled H_2_O) and lysed via sonication (Branson Sonifier 250 equipped with ½-inch titanium disruptor horn; Branson, Brookfield, Connecticut). Lysed biomass (3 mL) was extracted sequentially: first, mixed with 6 mL H_2_O and 9 mL hexane, vortexed (2 min), sonicated (bath, 15 min) and centrifuged (20,000 g × 5 min). Hexane and aqueous phases were discarded and pellets were resuspended in 12 mL acetonitrile:methanol (5:2 v/v), vortexed (2 min), sonicated (cooled bath, 1 h), centrifuged (20,000 g × 5 min), and the supernatant filtered (0.22 μM PTFE syringe filter) (Lech and Jarosz, 2011; Barrera Vázquez et al., 2014; Shi et al., 2014; Duval et al., 2016; Patel et al., 2019; Chia et al., 2020; Gurkok, 2022). For *P. marcusii* and *Cladosporium* sp. pigments were released in the liquid media during fermentation. After initial removal of cellular biomass via centrifugation, the cell-free supernatant (CFS, 10 mL) was extracted with 90 mL of ethanol, rested (5 min), centrifuged (20,000 g × 5 min), and the pigmented supernatant filtered as above (Shi et al., 2014; Duval et al., 2016; Patel et al., 2019). Extracts were analyzed by UPLC-QTOF-MS (Waters G2-XS QTOF, Milford, MA, United States) in ESI^–^ and full scan mode with a scan range m/z from 100 to 1,000 Da. Separation was performed with the following parameters. Acuity UPLC BEH C18 column (1.7 μm, 2.1 × 100 mm); mobile phases A (H_2_O + 0.5% NH_4_OH), and B (MeOH + 0.5% NH_4_OH). Gradient (flow 0.3 mL/min, injection 10 μL): 0–3 min 5% B, 3–6 min 25% B, 6–10 min 25% B, 10–16 min 66% B, 16–20 min 100% B, 20–21 min 5% B. Feature detection, blank filtering, and exact-mass matching ( ± 5 ppm against theoretical monoisotopic masses) were performed in UNIFI (v. 3.15.1) against a curated PubChem-derived list of candidate compounds (Kim et al., 2025).

## 3 Results

### 3.1 Isolation, taxonomic identification, and selection of pigmented strains

**FIGURE 1:**
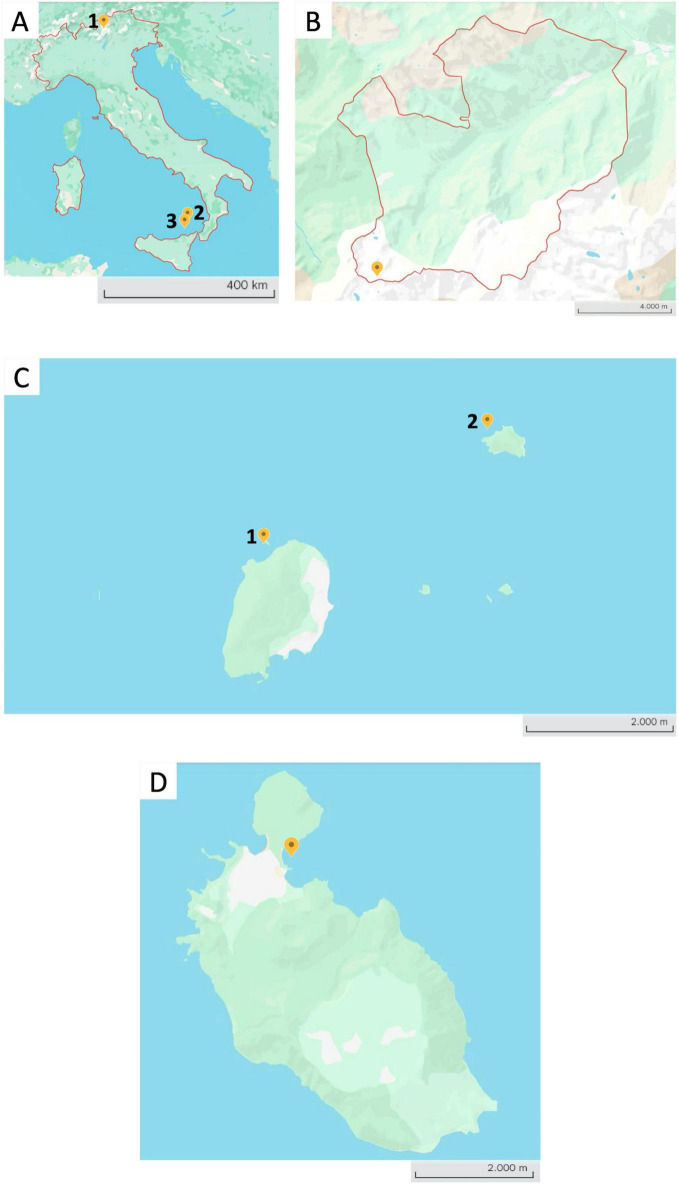
Map of sampling sites. **(A)** Location of the three sampling sites in Italy: site (1) Presena glacier; site (2) Panarea island; site (3) Vulcano island. **(B)** Specific location of sampling station within Presena glacier: “Passo presena” within the municipality of Vermiglio (highlighted as red area), Trentino-Alto Adige, Italy. **(C)** Specific location of the two sampling stations off the coast of Panarea island, Sicily, Italy. Station 1: underwater CO_2_ crater at 10 m depth; station 2: submerged cave located at a depth of 14 m off Basiluzzo Island. **(D)** Specific location of sampling station off the coast of Vulcano island, Italy.

**FIGURE 2:**
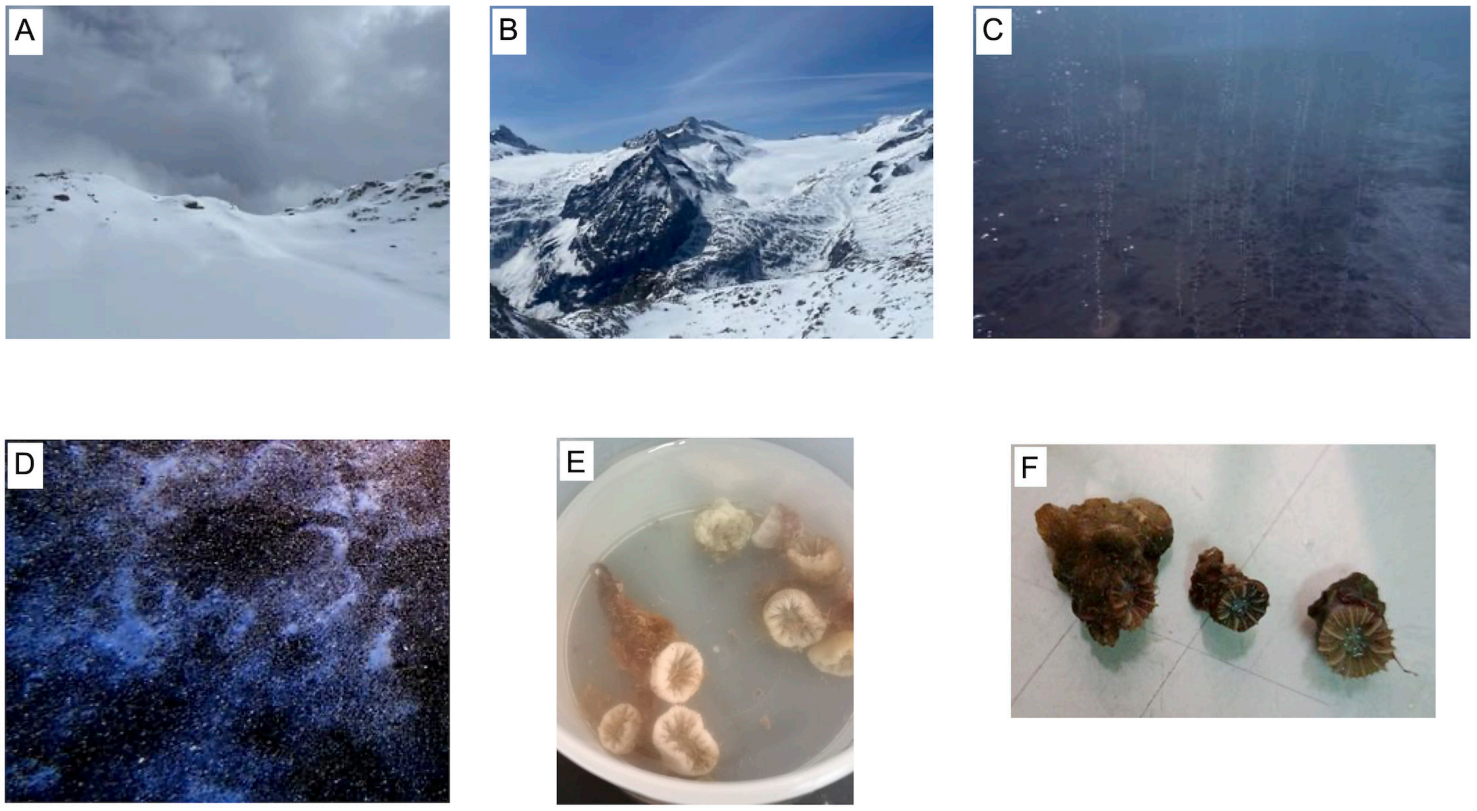
Overview of extreme environments sampled in this study as sources for the isolation of novel BNC-producing microorganism. **(A,B)** Presena Glacier, 3,000 m a.s.l., Trentino Alto Adige, Italy; **(C,D)** volcanic coastal marine sediments influenced by shallow hydrothermal CO_2_ rich venting from the volcanic island of Vulcano, Sicily, Italy; **(E)** picture of *Balanophyllia europaea* specimens retrieved from Panarea Island, Sicily, Italy; **(F)** picture of *Caryophylla inornata* specimens retrieved from Panarea Island, Sicily, Italy.

**FIGURE 3:**
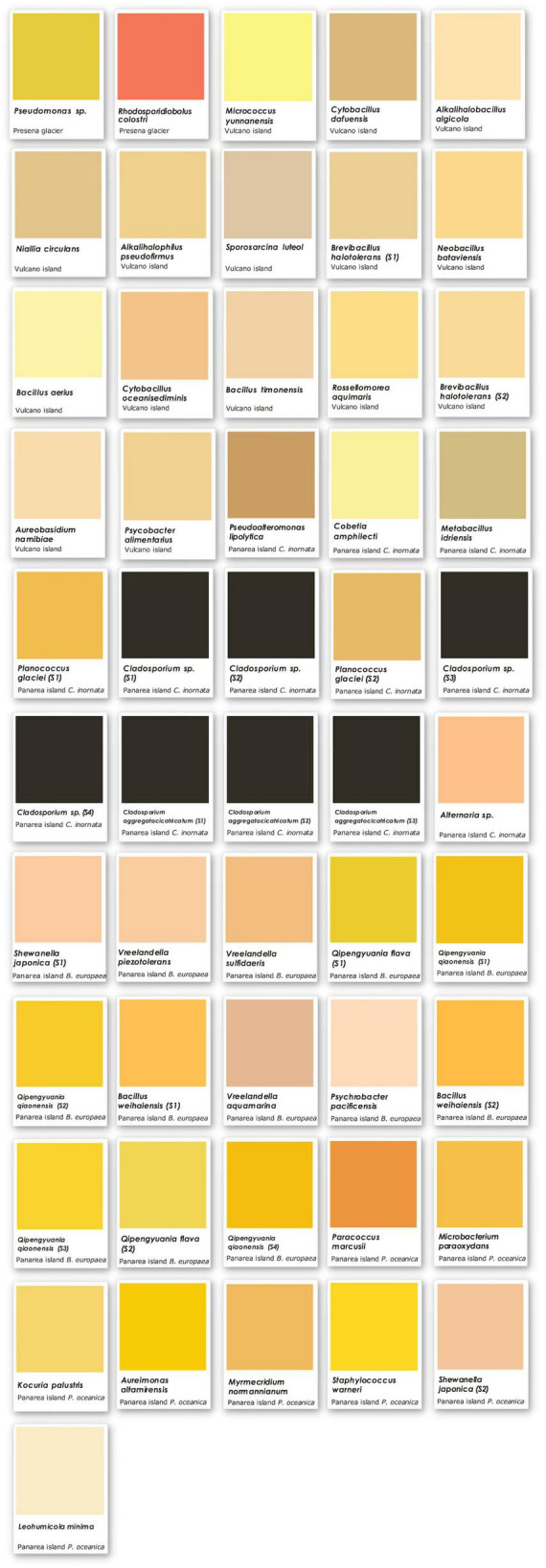
Chromatic diversity of the pigmented isolates. For each isolate, taxonomic classification, origin, and representative images of stationary-phase cellular pellets are provided.

Pigmented microbial strains with the potential for BNCs production were isolated from environmental and host-associated microbiomes collected across diverse natural ecosystems in Italy. The sampling sites and stations investigated in this study are reported in Figure 1. The selected sites encompass a range of ecologically distinct extreme habitats, including: the Presena Glacier, volcanic coastal marine sediments from Vulcano Island, and different marine holobionts (*B. europaea*, *C. inornata*, and *P. oceanica*) living in proximity to natural CO_2_ emission sources in Panarea Island (Figure 2). Two distinct pigmented colonies were successfully isolated from glacier samples; 15 from coastal volcanic marine sediments; 34 from marine holobionts, comprising 13 from *B. europaea*, 13 from *C. inornata*, and 8 from *P. oceanica*. These 51 isolates underwent molecular identification via 16S rRNA sequencing (bacteria) or ITS sequencing (fungi), as reported in Supplementary Table 1. Phylogenetic analysis based on 16S rRNA (bacteria) or ITS gene (fungi) sequences revealed that all pigment-producing isolates clustered closely with their respective reference taxa, confirming their taxonomic assignment across a broad range of bacterial and fungal genera (Supplementary Figure 2). In Figure 3 we provide the visual appearance of stationary-phase cellular pellets obtained from the identified pigmented isolates.

Pigmented isolates recovered from the glacier microbiome were identified as *Pseudomonas* sp. (yellow-pigmented bacterium) and *Rhodosporidiobolus colostri* (red-pigmented yeast). Among the isolates obtained from volcanic coastal marine sediments, 9 bacterial strains exhibiting a yellow colony phenotype were identified as *Cytobacillus dafuensis*, *Niallia circulans*, *Alkalihalophilus pseudofirmus*, *Sporosarcina luteola*, *Psychrobacter alimentarius*, *Neobacillus bataviensis*, *Bacillus timonensis*, *Micrococcus yunnanensis*, and *Bacillus aerius*. The remaining 6 isolates from the same environment displayed an orange pigmentation and were identified as *Alkalihalobacillus algicola*, *Cytobacillus oceanisediminis*, *Brevibacillus halotolerans* (two distinct strains), *Rossellomorea aquimaris*, and *Aureobasidium namibiae*. Among the 34 pigmented isolates recovered from host-associated microbiomes, 13 yellow-pigmented strains were annotated as *Cobetia amphilecti*, *Metabacillus idriensis*, *Planococcus glaciei*, *Kocuria palustris*, *Aureimonas altamirensis*, *Staphylococcus warneri*, *Qipengyuania flava*, and *Qipengyuania qiaonensis*; 9 orange-pigmented isolates were assigned to *Planococcus glaciei*, *Pseudoalteromonas lipolytica*, *Paracoccus marcusii*, *Microbacterium paraoxydans*, *Myrmecridium normannianum*, *Shewanella japonica*, *Vreelandella piezotolerans*, and *Vreelandella sulfidaeris*; 6 red-pigmented isolates were identified as *Bacillus weihaiensis*, *Vreelandella aquamarina*, *Psychrobacter pacificensis*, *Alternaria* sp., *Cladosporium* sp., and *Leohumicola* sp.; and 6 black-pigmented colonies were annotated as *Cladosporium* sp. and *Cladosporium aggregatocicaticatum*, both represented by multiple strains.

Eight pigmented isolates retrieved from *P. oceanica* were identified as *Paracoccus marcusii*, *Microbacterium paraoxydans*, *Myrmecridium normannianum*, *Shewanella japonica*, and *Leohumicola minima*, all exhibiting an orange colony phenotype. Yellow-pigmented isolates were assigned to *Kocuria palustris*, *Aureimonas altamirensis*, and *Staphylococcus warneri* (two distinct strains).

Overall, seven pigmented isolates, representing diverse environmental sources, displaying intense and stabile pigmentation and growing well in liquid culture (Supplementary Figure 1), were selected for downstream analyses. These comprised *Rhodosporidiobolus colostri* (glacier), *Cytobacillus oceanosediminis*, *Micrococcus yunnanensis*, and *Alkalihalobacillus algicola* (volcanic marine sediments), *Paracoccus marcusii* (*P. oceanica*-associated), and *Planococcus glaciei* and *Cladosporium* sp. (*C. inornata*-associated).

### 3.2 Genome assemblies, comparative genomics, BGC prediction and CAZymes profiles

Whole-genome sequences were obtained for the seven selected strains. Assembly statistics (length, completeness, contamination, largest contig, number of contigs, N50, L50, GC content, predicted ORF, coverage depth, ANI values and reference assembly) are provided in Table 1. Overall, the assembled genomes exhibited high quality, with completeness values ranging from 80 to 100% and low contamination levels (<1.3% in all cases). Most bacterial genomes showed high completeness (95–100%), low contamination, and relatively low numbers of contigs, indicating robust assemblies suitable for downstream genomic analyses. Although the fungal genomes were more fragmented, their completeness remained sufficiently high (98 and 80%, respectively) to support comparative genomic inference. For the sequenced strains we reconstructed the ANI distance profiles against NCBI RefSeq/GenBank reference genomes (Supplementary Figure 3). ANI values were: *P. marcusii* 97.58% (vs. ASM2862171v1); *M. yunnanensis* 98.06% (vs. ASM5297846v1); *P. glaciei* 98.40% (vs. ASM1336877v1). Interestingly, the genomes of *A. algicola* and *C. oceanosediminis* displayed ANI distance values below the 95% (the species delineation threshold) when compared with their closest references, suggesting that these isolates may represent novel genomic lineages within their respective species. Regarding the fungal isolates, *R. colostri* showed 99.05% ANI distance with the reference genome ASM5347719v1, and *Cladosporium* sp. isolate exhibited an ANI distance of 95.98% relative to *Cladosporium allicinum* ASM5257632v1, supporting its taxonomic placement and complementing the ITS-based identification. For *C. oceanisediminis* and *M. yunnanensis*, for which more than 20 genomes were available in public databases, an in-depth comparative genomic assessment was performed (Supplementary Figure 4). The *M. yunnanensis* isolate displayed an overall genomic profile largely consistent with those of previously sequenced strains. In contrast, the *C. oceanisediminis* isolate exhibited a markedly distinct genomic architecture compared with publicly available genomes, suggesting a substantial degree of genomic novelty. This pattern was consistently supported by the PCoA, Neighbor-Joining, and UPGMA analyses, in which the isolate from the present study formed a clearly differentiated cluster relative to the currently available *C. oceanisediminis* genomes, highlighting

**TABLE 1:** Whole-genome sequences of assembly statistics for the seven selected microbial strains.

Isolate	Length	Completeness	Contamination	Largest contig	Number of contigs	N50	L50	GC content	Number of ORFs	Coverage depth	ANI Value	Assembly
*Rhodosporidiobolus colostri*	24.41 Mb	98%	0.30%	227,999 b	1244	43,781 b	165	59.90%	8,756	56	99.05%	ASM5347719v1
*Cytobacillus oceanisediminis*	4.8 Mb	87%	0.64%	707,829 b	35	515,084 b	4	46.01%	4,919	153	71.58%	ASM783023v1
*Alkalihalobacillus algicola*	4.1 Mb	100%	0.40%	308,061 b	50	187,244 b	9	40.58%	4,360	154	94.57%	ASM2017180v1
*Micrococcus yunnanensis*	2.4 Mb	96%	0.00%	362,215 b	42	268,519 b	4	73.12%	2,223	328	98.06%	ASM5297846v1
*Paracoccus marcusii*	4.2 Mb	100%	0.91%	1,121,956 b	91	265,798 b	4	66.10%	4,069	158	97.58%	ASM2862171v1
*Cladosporium* sp. (strain 1)	25.1 Mb	80%	0.20%	208,515 b	1060	50,247 b	165	53.71%	9,204	45	95.98%	ASM5257632v1
*Planococcus glaciei* (strain 1)	3.9 Mb	95%	1.24%	1,305,237 b	19	849,506 b	2	46.78%	3,892	255	98.40%	ASM1336877v1

Genome assembly statistics for the seven pigmented microbial isolates are reported. Metrics include assembly length, completeness, contamination, largest contig, number of contigs, N50, L50, GC content, predicted ORFs, sequencing coverage depth, ANI values and reference assembly. For organisms that have been isolated multiple times, selected strains are reported in brackets. ORFs, Open Reading Frames.

its unique genomic features. In order to profile the biosynthetic potential of BNC for the selected taxa, the sequenced genomes were analyzed for their BGCs content with antiSMASH (Table 2). We identified five BGCs in *R. colostri*, three classified as terpene-related, and two as NRPS-like clusters. Among the terpene-related BGCs, one was assigned to ascofuranone biosynthesis (a yellow pigmented compound), one to carotenoid biosynthesis, and one unassigned. Five BGCs were identified for *C. oceanosediminis*, three classified as terpene-related, one as a Type (T) 3 PKS cluster, and one as a class II lanthipeptide cluster. Among the terpene-related, one was putatively assigned to alkylresorcinol biosynthesis, one to carotenoid biosynthesis, one unassigned. The T3PKS cluster was predicted to be involved in fumihopaside A biosynthesis. For *M. yunnannensis*, we identified a heterogeneous repertoire of 6 BGCs spanning distinct families. These comprised one cluster assigned to ectoine biosynthesis, one RRE-containing cluster, one β-lactone cluster, one terpene cluster, one NAPAA-class cluster, and one Ni-siderophore cluster. *A. algicola* harbored 6 BGCs, two terpene-related clusters (fumihopaside A biosynthesis and production of a carotenoid-like compound), one β-lactone cluster, one Ni-containing siderophore cluster, one RRE-containing cluster, and one T3PKS cluster (biosynthesis of an alkylresorcinol). We identified nine BGCs in *P. marcusii*, a terpene cluster (putatively assigned to the biosynthesis of a carotenoid), T3PKS (alkylresorcinol production), and a hybrid NRPS–PKS cluster (biosynthesis of an obafluorin-like compound). In *P. glacei*, 4 BGCs were identified: one T3PKS cluster (alkylresorcinol biosynthesis), and three terpene clusters (production of carotenoid-like compounds). Analysis of the *Cladosporium* sp. genome revealed a remarkable repertoire of 26 BGCs. Seven of these were associated with classes potentially involved in pigment production, encompassin two T1PKS clusters, three terpene clusters, and two hybrid NRPS–T1PKS clusters. More specifically, the first T1PKS cluster was associated with the putative production of a betaenone-like compound. The second T1PKS cluster showed similarity to 1,3,6,8-tetrahydroxynaphthalene biosynthetic clusters, suggesting the potential production of naphthalene-type molecules. Among the hybrid NRPS–T1PKS clusters, one was predicted to direct the biosynthesis of a tetralin-derived compound, whereas the second could not be confidently linked to a specific product. Finally, of the three terpene clusters, one could not be assigned to any known metabolite; the second was associated with the putative production of a fumihopaside-like compound, and the third was linked to the biosynthesis of a carotenoid-type molecule. To assess the degree of BGC novelty characterizing the isolate genomes, the predicted BGC repertoires were compared with those identified in the corresponding reference genomes (Supplementary Table 2). This comparative analysis revealed that *Cladosporium* sp., *R. colostri*, *C. oceanisediminis*, and *P. marcusii* exhibit BGC profiles that differ substantially from those of their respective reference genomes, indicating a degree of novelty in their biosynthetic potential. These differences suggest the presence of unique secondary metabolite biosynthetic capabilities. Finally, a CAZyme family profile was generated for each isolate (Supplementary Figure 5). The results revealed a highly heterogeneous glycobiome architecture across the different isolates. Among the fungal strains, *Cladosporium* sp. and *R. colostri* exhibited the greatest CAZyme diversity, with 74 and 64 annotated CAZyme families, respectively, including 32 and 27 glycoside hydrolase (GH) families. In contrast, the bacterial isolates generally displayed a lower, yet still substantial, glycobiome diversity. Among them, *C. oceanosediminis* showed the richest CAZyme repertoire, harboring 43 CAZyme families, including 19 GH families. *P. marcusii*, *A. algicola*, and *P. glacei* possessed 35, 33, and 30 CAZyme families, respectively, of which 13, 12, and 14 belonged to the GH class. *M. yunnanensis* exhibited the lowest glycobiome diversity, with only 14 CAZyme families identified, including 6 GH families.

**TABLE 2:** Biosynthetic potential of bioactive natural compounds production for the selected strains.

Selected isolate	Region	Start position	End position	Total length (nt)	AntiSMASH class	Associated product	Score	Most similar cluster
*Rhodosporidiobolus colostri*	33.1	1	24,274	24,274	Terpene	Ascofuranone	0.23	BGC0001924
	73.1	36,059	69,288	33,230	NRPS-like	Myxochelin	0.09	BGC0002324
	229.1	1	18,145	18,145	Terpene	Carotenoid	0.32	BGC0000647
	234.1	1	20,121	20,121	Terpene	/	/	/
	343.1	1	24,319	24,319	NRPS-like	Livipeptin	0.20	BGC0001168
*Cytobacillus oceanisediminis*	1.1	119,735	140,553	20,819	Terpene	/	/	/
	2.1	127,642	168,727	41,086	T3PKS	Alkylresorcinol	0.34	BGC0000282
	5.1	46,557	75,614	29,058	Lanthipeptide	SmbA	0.34	BGC0000552
	7.1	5,571	76,606	20,897	Terpene	Carotenoid	0.36	BGC0000647
	19.1	10,218	32,104	21,887	Terpene	Fumihopaside A	0.24	BGC0002173
*Micrococcus yunnanensis*	3.1	73,315	83,686	10,372	Ectoine	Ectoine	0.31	BGC0002405
	4.1	49,547	69,879	20,333	RRE-containing	Tetrabromopyrrole	0.07	BGC0001464
	5.1	87,476	114,353	26,878	betalactone	Deoxyhangtaimycin	0.24	BGC0002657
	8.1	109,342	130,238	20,897	Terpene	Carotenoid	0.35	BGC0000644
	12.1	643	3,465	34,008	NAPAA	ε-Poly-L-lysine	0.38	BGC0002174
	15.1	1	21,566	21,566	NI-siderophore	Desferrioxamine	0.50	BGC0001478
*Alkalihalobacillus algicola*	1.1	15,105	171,886	20,837	Terpene	Carotenoid	0.41	BGC0000645
	4.1	209,091	229,351	20,261	RRE-containg	Exopolisaccaride	0.25	BGC0000742
	7.1	170,849	192,669	21,821	Terpene	Fumihopaside A	0.23	BGC0002173
	18.1	10,084	39,419	29,336	Betalactone	Fosfomycin	0.24	BGC0001859
	26.1	22,232	45,182	22,951	Ni-siderophore	Vibrioferrin	0.36	BGC0002527
	37.1	1	15,958	15,958	T3PKS	Alkylresorcinol	0.35	BGC0000282
*Paracoccus marcusii*	1.1	276,892	302,832	25,941	Betalactone	Corynecin	0.22	BGC0002284
	1.2	757,803	795,062	37,260	Ectoine	Ectoine	0.91	BGC0000860
	2.1	144,827	167,002	22,176	RiPP	Aborycin	0.32	BGC0002285
	3.1	351,388	374,961	23,574	Terpene	Carotenoid	0.88	BGC0000635
	3.2	392,446	431,934	39,489	T3PKS	Alkylresorcinol	0.34	BGC0000282
	7.1	48,874	70,115	21,242	RRE-containing	Exopolisaccaride	0.20	BGC0000756
	7.2	140,77	151,615	10,846	RiPP-like	Pseudopyronine	0.12	BGC0001285
	7.3	151,631	228,287	76,657	Hybrid NRPS-T1PKS	Pyreudione	0.95	BGC0002075
	14.1	6,674	27,264	20,591	Homoserine lactone	Carnocyclin	0.18	BGC0000487
*Planococcus glaciei* (strain 1)	2.1	737,259	758,092	20,834	Terpene	Carotenoid	0.52	BGC0000645
	3.1	205,564	246,634	41,071	T3PKS	Alkylresorcinol	0.29	BGC0000282
	3.2	356,613	377,443	20,831	Terpene	(2R,3S,4S)-5-fluoro-2,3,4-trihydroxypentanoic acid	0.33	BGC0001928
	5.1	200,728	221,585	20,858	Terpene	Carotenoid	0.33	BGC0000647
*Cladosporium* sp. (strain 1)	7.1	111,984	142,252	30,269	NRPS-like	Livipeptin	0.20	BGC0001168
	8.1	96,026	139,532	43,507	NRPS-like	Choline	0.26	BGC0002276
	9.1	1	26,323	26,323	T1PKS	Betaenone	0.21	BGC0001264
	9.2	43,504	10,672	63,217	NRP-metallophore	Epichloenin	0.61	BGC0001250
	15.1	1	100,734	100,734	NRPS	Cyclic tetrapeptide	0.29	BGC0000357
	18.1	1,828	63,541	45,262	NRPS-like	Asperipin 2a	0.16	BGC0001306
	22.1	53,742	87,417	33,676	NAPAA	Betaestacin	0.36	BGC0001528
	24.1	945	46,551	45,607	NRPS	Ferrichrome	0.42	BGC0000900
	26.1	1	29,205	29,205	NRPS-like	Choline	0.45	BGC0002276
	36.1	1	32,625	32,625	NRPS	Tacrolimus	0.09	BGC0000353
	49.1	6,235	82,487	76,253	Hybrid NRPS-T1PKS	Burnettiene	0.70	BGC0002139
	59.1	38,769	76,811	38,043	NRPS-like	Orsellinic acid	0.11	BGC0002212
	62.1	1	75,766	75,766	NRPS	Asperphenamate	0.35	BGC0001517
	67.1	1	1,796	17,960	Indole	Haloduracin	0.13	BGC0000517
	128.1	1	30,238	30,238	NRPS-like	Aspulvinone E	0.33	BGC0002348
	147.1	1	16,244	16,244	Terpene	Carotenoid	0.32	BGC0000647
	166.1	8,917	50,232	41,316	NRPS-like	Fosfonochlorin	0.29	BGC0002670
	213.1	1	4,359	43,590	Hybrid NRPS-T1PKS	Depudecin	0.52	BGC0000046
	220.1	1	34,909	34,909	NRPS	Tenuazonic acid	0.19	BGC0002158
	284.1	1	33,725	33,725	NRPS	Surfactin	0.06	BGC0000433
	301.1	9,196	33,476	24,281	betalactone	Corynecin	0.17	BGC0002284
	335.1	5,760	29,895	24,136	NRPS	Kolossin	0.20	BGC0001641
	361.1	1	26,852	26,852	NRPS-like	Nidulanin A	0.17	BGC0001699
	525.1	1	12,293	12,293	Terpene	Fumihopaside A	0.18	BGC0002173
	592.1	1	8,685	8,685	Terpene	/	/	/
	633.1	1	664	6,640	T1PKS	1,3,6,8-tetrahydroxynaphthalene	0.20	BGC0001258

AntiSMASH results are reported for each isolated strain (column 1). Columns 2–5 refer to the genomic region where the biosynthetic gene cluster (BGC) is found. Columns 6–9 report to the BGC class as predicted by AntiSMASH, associated products and most similar clusters, with the respective score, deposited in MIBiG.

### 3.3 Carbohydrate utilization profile and growth kinetics

Carbohydrate metabolism was assessed for the seven selected stains using API 50 Ch strips under aerobic conditions (Supplementary Table 3). According to our findings, *R. colostri* showed the capacity to catabolize different monosaccharides (namely, galactose, glucose, fructose, and mannose), several phenolic glycosidic compounds (i.e., amygdalin, esculin, and salicin), as well as the disaccharides maltose, lactose and sucrose. Similarly, *C. oceanosediminis* utilized various monosaccharides, including galactose, glucose, and fructose, phenolic glycosidic compounds, as well as several disaccharides, such as cellobiose, maltose, lactose, melibiose, and sucrose, and the plant-derived trisaccharide raffinose and starch. These findings indicate a broad *in vitro* capacity to degrade both amylaceous and cellulosic substrates, such as plant-associated carbohydrates and glycosides. Conversely, *M. yunnannensis* carbohydrate metabolism was limited to few disaccharides, such as maltose, sucrose, and trehalose. *A. algicola* utilized monosaccharides (galactose, glucose, fructose, and mannose), disaccharides (maltose, sucrose, and trehalose), the plant-derived trisaccharide melezitose, as well as starch and glycogen, indicating the potential to catabolize amylaceous substrates and plant carbohydrates. *P. marcusii* showed the broadest versatility, being able to metabolize several monosaccharides, both pentoses and hexoses, as well as plant-derived phenolic glycosides. In addition, *P. marcusii* utilized disaccharides and trisaccharides of plant origin, starch, and glycogen. Finally, *Cladosporium* was able to catabolize several plant-derived glycosidic phenolic compounds, including amygdalin, arbutin, esculin, and salicin, disaccharides, such as melibiose and trehalose, as well as the plant-derived trisaccharides melezitose and raffinose. These findings further support the capacity of *Cladosporium* to utilize structurally diverse plant-associated carbohydrates and glycosides under in vitro conditions. The observed ability of *R. colostri*, *C. oceanosediminis*, *A. algicola*, *P. marcusii*, and *Cladosporium* sp. strains to metabolize plant-derived carbohydrates is consistent with the diversity of CAZyme families, particularly glycoside hydrolases (GHs), identified in their genomes.

For six out of the seven selected strains, batch culture growth parameters were obtained (Table 3). For *R. colostri*, the specific growth rate (μ) in TBS medium at 20°C was 0.35 h^−1^, with a carrying capacity (K) of 7.28 absorbance units (AU). The estimated doubling time (t_d) was 1 h 59 min. For *C. oceanosediminis*, the μ in TBS medium at 30°C was 0.39 h^−1^, with a K of 3.87 AU, and estimated d_t 1 h 47 min. *M. yunnannensis* showed a μ of 0.3 h^−1^ (TBS, 30°C), with K = 19.7 AU and d_t = 2 h and 17 min. *P. glacei* growth parameters were μ = 0.31 h^−1^ (MB, 25°C), K = 8.67 AU, d = t = 2 h and 16 min. Kinetic parameters for *A. algicola* (MB, 30°C) were μ = 0.28 h^−1^, K = 7.28 AU, d_t = 2 h and 31 min. *P. marcusii* showed μ = 0.37 h^−1^ (MB, 25°C), K = 6.75 AU, and d_t = 1 h and 53 min.

**TABLE 3:** Batch culture growth parameters of the selected strains.

Isolate	Growth rate (μ)	Carrying capacity (K)	Doubling time	Temperature	Media
*Rhodosporidiobolus colostri*	0.35 h^−1^	7.28	1 h 59 min	20°C	TSB
*Cytobacillus oceanisediminis*	0.39 h^−1^	3.87	1 h 47 min	30°C	TSB
*Alkalihalobacillus algicola*	0.28 h^−1^	7.28	2 h 31 min	30°C	MB
*Micrococcus yunnanensis*	0.30 h^−1^	19.7	2 h 17 min	30°C	TSB
*Paracoccus marcusii*	0.37 h^−1^	6.75	1 h 53 min	25°C	MB
*Planococcus glaciei* (strain 1)	0.31 h^−1^	8.67	2 h 16 min	25°C	MB

Kinetic parameters of specific growth rate (μ), carrying capacity (K), and doubling time were obtained from fitting each strain growth on a logistic curve model. Carrying capacity is reported as an absorbance value. Abbreviations: TSB = triptone soya broth; MB = marine broth.

### 3.4 UPLC-MS profiles of secondary metabolites

UPLC-MS profiling of batch fermentation extracts revealed distinct secondary metabolites production potential across selected strains (Table 4). For *R. colostri* we detected antheraxanthin, torularhodin, adonirubin, and 2-hydroxytorularhodin, compounds belonging to the carotenoid family, a class of pigments responsible for yellow, orange, and red coloration, consistent with the characteristic pigmentation observed in this strain. *C. oceanosediminis* analysis detected (2R, 3S, 3’S)-2-hydroxy astaxanthin, echinenone, canthaxanthin (carotenoids), chrysophanol (anthraquinone), macrolactin H, and bacillaene. The presence of multiple carotenoids indicates an active carotenoid biosynthetic pathway, explaining the orange–red pigmentation observed in the biomass or crude extracts. In addition, chrysophanol may contribute to yellow–orange tones, potentially modulating the overall coloration when co-produced with carotenoids. *A. algicola* extracts analysis revealed the presence of chrysophanol, bacillaene, macrolactin H, surfactin A, 7-deoxypactamycin, difficidin, and bacilysin. In *P. marcusii* extracts, we detected ethyl troposulfenin, roseobacticide, paracentrone, tryptanthrin, rhamnolipid, adonirubin, 2-hydroxyastaxanthin, adonixanthin 3-glucoside, and adonixanthin, with the last four compounds belonging to the xanthophyll subclass of carotenoids and carrying extended conjugated double-bond systems, which absorb visible light and confer yellow, orange, or red coloration. *P. glacei* analysis detected histidyl-proline diketopiperazine, lisinopril R,S,S-diketopiperazine, the glycosylated apocarotenoid methyl 5-glucosyl-5,6-dihydro-apo-4,4’-lycopenoate, rhamnolipid, and decaprenoxanthin. The detection of both an apocarotenoid and decaprenoxanthin provides evidence of an active carotenoid biosynthetic pathway in *P. glacei*, supporting its yellow to orange coloration. For *M. yunnannensis* several secondary metabolites were detected, including carotenoids (2R, 3S, 3’S)-2-hydroxy astaxanthin, echinenone, adonirubin, and paracentrone, as well as several antimicrobial peptides, such as N-acetyldehydro-phe-pro diketopiperazine, phenylalanyl-prolyl diketopiperazine, histidyl-proline diketopiperazine, and the biosurfactant rhamnolipid. Finally, extracts of *Cladosporium* sp. revealed a remarkable diversity of secondary metabolites, including isocladosporin, cladosporin, endocrocin, 1,8-dihydroxynaphthalene, cladosacid, cladospolide, cladofulvin, 2-(4-hydroxy-1,3-dihydro-2-benzofuran-1-yl) acetic acid, 3-phenyllactic acid, cladosin, cladosporol, calphostin, cladochrome, haematocin, cladodionen, and cladosporiumin. Among these, several compounds (i.e., endocrocin, cladofulvin, and cladochrome) are polyketide-derived pigments (including anthraquinones and possibly melanin precursors) consistent with the well-known chromogenic potential of *Cladosporium* species.

**TABLE 4:** Secondary metabolites production during batch fermentation of the selected strains.

Isolate	Name	Mass
*Rhodosporidiobolus colostri*	Canthaxanthin	564.80
	Adonirubin	580.80
	Torularhodin	564.80
	2-hydroxytorularhodin	580.80
*Cytobacillus ocenosediminis*	Chysophanol	254.24
	Canthaxanthin	564.80
	(2R, 3S, 3S)-2-hydroxyastaxanthin	612.80
	Bacillaene	580.80
	Macrolactin H	402.50
	Echinenone	550.90
*Alkalihalobacillus algicola*	7-Deoxypactamycin	542.60
	Difficidin	544.70
	Bacillaene	580.80
	Macrolactin H	402.50
	Surfactin A	1007.65
	Bacilysin	270.28
	Chysophanol	254.24
*Micococcus yunnanensis*	(2R, 3S, 3S)-2-hydroxyastaxanthin	612.80
	Echinenone	550.90
	Adonirubin	580.80
	Paracentrone	462.70
	N-Acetyldehydro-phe-pro diketopiperazine	284.31
	Phenylalanyl-prolyl diketopiperazine	244.29
	Histidyl-proline diketopiperazine	248.28
	Ramonolipid C10	334.40
*Paracoccus marcusii*	Adonirubin	580.80
	Paracentrone	462.70
	Tryptanthrin	248.24
	Roseobacticide H	362.40
	Methyl troposulfenin	226.30
	(2R, 3S, 3S)-2-hydroxyastaxanthin	612.80
	Adonixanthin 3-glucoside	745.00
	Adonixanthin	582.90
	1-Tridecanol	398.70
	Roseobacticide C	307.40
	Rhamnolipid R1	650.80
*Cladosporium* sp. (strain 1)	Calphostin	790.80
	Cladochrome	774.80
	Cladosporol	350.30
	Isocladosporin	292.33
	Cladosporin	292.33
	Cladosporide	386.60
	Cladosin	282.34
	Cladosacid	250.33
	Endocrocin	314.25
	1,8-dihydroxynaphthalene	160.17
	Cladofulvin	538.50
	(+-)-3-Phenyllactic acid	166.17
	2-(4-hydroxy-1,3-dihydro-2-benzofuran-1-yl) acetic acid	194.18
	Cladospolide	228.28
	Haematocin	502.60
	Cladodionen	233.26
	Cladosporiumin	349.40
	Malettinin	288.29
*Planococcus glaciei* (strain 1)	Decaprenoxanthin	705.10
	5-glucosyl-5,6-dihydro-4,4′-diapolycopenene	766.30
	Histidyl-proline diketopiperazine	248.28
	Lisinopril R,S,S-diketopiperazine	387.50

Column two reports for all compounds identified via UPLC-MS for each strain. Column three reports mass values for the compounds as measured by Mass detector.

## 4 Discussion

In our work, we successfully isolated, identified, and characterized BNC-producing microorganisms from diverse natural ecosystems such as, a glacier, volcanic coastal marine sediments and different marine holobionts (*B. europaea*, *C. inornata*, and *P. oceanica*) living in proximity to natural CO_2_ emission sources. These ecosystems were selected as representative contexts favoring microbial with BNCs producing capabilities (Tyc et al., 2017). To enrich microbial taxa able to produce BNCs also in laboratory condition, a pigmentation-guided isolation strategy was employed. Our approach resulted in the isolation of 51 pigmented microorganisms (39 bacteria and 12 fungi) from five natural microbiomes, including a glacier ecosystem, volcanic coastal marine sediments, and host-associated microbiomes from corals (*B. europaea* and *C. inornata*) and the seagrass *P. oceanica*. Overall, the pigmented isolates displayed a substantial phylogenetic diversity and were distributed across three major phyla. Among bacteria, 14 isolates belonged to *Bacillota*, 8 to *Pseudomonadota*, and 4 to *Actinomycetota*. Fungal isolates were affiliated with two phyla, with 7 strains assigned to *Ascomycota* and 1 strain to *Basidiomycota*. The extensive phylogenetic diversity of the pigment-producing isolates underscores the widespread occurrence of pigment biosynthesis across bacterial and fungal taxa. This finding suggests that pigment production represents a common adaptive ecological trait in both environmental and host-associated microbiomes, rather than being restricted to particular evolutionary lineages or ecological niches. Seven pigmented isolates, representative of diverse isolation sources and colony colors, and exhibiting stable growth and consistent pigmentation in liquid culture, were selected for in-depth bioprospecting as candidate microbial cell factories for the sustainable production of pigments and bioactive compounds.

From the Presena Glacier, one red-pigmented isolate was selected for downstream analysis, identified as *Rhodosporidiobolus colostri*. Genomic assessment placed the isolate within the same clade as the reference strain *R. colostri* ASM5347719, with an ANI value of 99.05%. *R. colostri* is a cold-adapted, carotenoid-producing basidiomycetous yeast commonly associated with soil and plant-related habitats and capable of persisting in exposed, low-temperature environments such as alpine snow (Turchetti et al., 2018; Urbina and Aime, 2018). *R. colostri* whole genome sequencing revealed the presence of five BGCs, two of which were annotated as carotenoid-biosynthetic clusters. Consistent with the genome mining results, batch fermentation experiments demonstrated that this strain produces multiple carotenoids, including antheraxanthin, torularhodin, adonirubin, and 2-hydroxytorularhodin. These yellow-orange pigments are known to exert important biological functions, including photoprotection and resistance to oxidative stress (Sakaki et al., 2000; Sakaki et al., 2001; Maoka et al., 2013). Such functions are likely advantageous for survival in glacier ecosystems, where microorganisms are exposed to high levels of UV-radiation, low temperatures, and oxidative stress associated with freeze-thaw cycles (Rekadwad et al., 2024; Crosta et al., 2025). Beyond their ecological relevance, these compounds represent bio-based colorants with promising bioprospecting potential. Microbial carotenoids are increasingly investigated as sustainable alternatives to synthetic pigments for industrial applications, including dyes, food additives,

and cosmetics (Pereira Da Costa and Campos Miranda-Filho, 2020; Dasgupta Mandal and Majumdar, 2023). In this context, the *R. colostri* isolate demonstrated an impressive CAZymes diversity, as well as the ability to catabolize plant-derived phenolic compounds and plant disaccharides. Besides being consistent with adaptation to litter decomposition in alpine soils, these metabolic capabilities also support the potential of this strain as a candidate for sustainable biotechnological production processes, particularly in the valorization of plant-derived substrates and agro-industrial residues.

From volcanic coastal marine sediments, three pigmented microbial isolates were selected for in-depth bioprospecting. The first isolate, characterized by intense yellow pigmentation, was identified as *Micrococcus yunnanensis* and clustered within the same phylogenetic clade as the reference strain ASM529784. *M. yunnanensis* is a Gram-positive, aerobic actinobacterium belonging to the genus *Micrococcus*, typically isolated from diverse environmental habitats and known for its tolerance to stress conditions (Rasuk et al., 2017; Aroumougame, 2026). Whole genome sequencing of the isolate revealed six BGCs, three of which were associated with known BNC classes: (i) ectoine, known to confer protection against osmotic, oxidative, and thermal stress (Kunte et al., 2014); (ii) a β-lactone (quorum sensing related molecule); and (iii) a Ni-siderophore The capacity to produce Ni-binding siderophores may enhance the fitness of *M. yunnanensis* in volcanic coastal marine sediments by regulating nickel bioavailability, facilitating its acquisition as an essential micronutrient, and reducing metal toxicity through sequestration under high-nickel conditions. Consistent with genome mining predictions, metabolomic profiling of the batch fermentation broth revealed the presence of several secondary metabolites, including multiple carotenoids (zeaxanthin, astaxanthin, echinenone, adonirubin, adonixanthin, and paracentrone) and antimicrobial peptides (N-acetyldehydro-phe-pro diketopiperazine, phenylalanyl-prolyl diketopiperazine, and histidyl-proline diketopiperazine). The second isolate, exhibiting orange pigmentation, was identified as *Alkalihalobacillus algicola*. Notably, this isolate displayed an ANI value below the 95% threshold, suggesting that these isolates may represent novel genomic lineages. *A. algicola* is a Gram-positive, spore-forming, aerobic bacterium within the family *Bacillaceae*, typically associated with marine or saline environments and adapted to alkaline conditions (Ivanova et al., 2004; Joshi et al., 2022). Genome sequencing of this novel isolate revealed five distinct BGCs associated with several BNCs, including fumihopaside A, a triterpenoid glycoside reported to confer resistance to UV radiation and high temperature (Seo et al., 2024), carotenoids, alkylresorcinol, an antimicrobial-PKS (Kanda et al., 1975; Gadea et al., 2022), and a siderophore. Batch fermentation experiments confirmed the production of multiple bioactive secondary metabolites, including compounds with reported antibacterial, antiviral, and anti-inflammatory activities. Among these were the polyketide-derived macrolactin H, difficidin, and 7-deoxypactamycin, as well as the nonribosomal antimicrobial dipeptide bacilysin (Fazle Rabbee and Baek, 2020). Additionally, surfactin A, an NRPS-derived biosurfactant, and chrysophanol, a yellow orange anthraquinone-type polyketide with antioxidant properties (Shi et al., 2014; Duval et al., 2016), were detected. Finally, the third isolate from volcanic marine sediments, also exhibiting orange pigmentation, was assigned to *Cytobacillus oceanisediminis*. This strain displayed an ANI value below 95% compared with available reference genome, indicating a potentially novel genomic lineage within the species. Comparative genomic analyses revealed that this newly isolated *C. oceanisediminis* strain harbors substantial genomic novelty relative to currently available genomes. The marked genomic divergence observed in the isolate initially assigned to *C. oceanisediminis* suggests that it may represent a distinct genomic lineage and potentially belong to a different species, highlighting the need for further taxonomic investigation. *C. oceanisediminis* is a Gram-positive, rod-shaped, spore-forming, aerobic bacterium originally isolated from marine sediments inhabiting mesophilic and saline benthic environments (Zhang et al., 2010). Genome mining of this new isolate identified five BGCs associated with three main BNC classes: carotenoids, fumihopaside A, and alkylresorcinol, a phenolic polyketide with antimicrobial properties (Kanda et al., 1975; Gadea et al., 2022). Batch fermentation experiments confirmed the production of several secondary metabolites of potential industrial relevance. These included: (i) the carotenoids echinenone and canthaxanthin, yellow-orange pigments known for their antioxidant and photoprotective properties; (ii) antimicrobial polyketides such as macrolactin H and bacillaene (Karpiñski, 2019; Ortiz and Sansinenea, 2020; Miao et al., 2023; Pedroza-Padilla et al., 2025; Vasilchenko et al., 2025); and (iii) chrysophanol, an anthraquinone-type polyketide characterized by yellow-orange pigmentation and antioxidant activity (Shi et al., 2014; Duval et al., 2016). Taken together, our findings indicate that isolates from volcanic coastal marine sediments possess the capacity to synthesize a diverse portfolio of BNCs, including pigments, antimicrobial molecules, and antioxidants metabolites. Such metabolic versatility likely represents an adaptive response to the physiochemical characteristic of volcanic sediments’ environments. At the same time, the production of these bioactive compounds highlights the promising biotechnological potential of these microorganisms. In particular, the putative novel lineages identified within the *A. algicola* and *C. oceanisediminis* demonstrated a relevant glycobiome diversity, as well as the ability to metabolize amylaceous substrates and plant-derived carbohydrates. Besides conferring a selective advantage in marine sediments through the exploitation of the diverse carbon sources available within the sediment matrix, these catabolic capabilities also highlight the potential of these strains for sustainable biotechnological applications, particularly processes based on the conversion of renewable feedstocks into value-added products.

Selected isolates from host-associated microbiomes included the orange-pigmented *Planococcus glaciei* and *Paracoccus marcusii*, the former recovered from *C. inornata* and the latter from *Posidonia oceanica*, as well as the black-pigmented *Cladosporium* sp., isolated from *C. inornata*. Genomic analysis showed that the three strains clustered within the same clades as their respective reference genomes, namely ASM1336877 (98.40% ANI), ASM2862171 (97.58% ANI), and ASM5257632 (95.98% ANI), the latter corresponding to *Cladosporium allicinum*. *P. glaciei* is an aerobic Gram-positive bacterium that, to the best of our knowledge, has not previously been reported from coral tissues. Genome mining of the sequenced isolate predicted four BGCs, associated with the production of carotenoid compounds and the antimicrobial polyketide alkylresorcinol (Kanda et al., 1975; Gadea et al., 2022). Metabolomic analysis of the batch fermentation culture revealed the production of several yellow-orange carotenoids, including methyl 5-glucosyl-5,6-dihydro-apo-4,4′-lycopenoate and decaprenoxanthin. In addition, antimicrobial compounds were detected, such as the quorum-sensing disruptor histidyl-proline diketopiperazine (NRPS-derived) and the biosurfactant rhamnolipid (Chen et al., 2017; Zink et al., 2021; Bailly, 2025). Conversely, *P. marcusii* is a pigment-producing marine heterotroph previously isolated from marine crustaceans (Leinberger et al., 2021), although, to the best of our knowledge, it has not been reported from seagrass tissues. Our findings revealed broad biosynthetic potential for this strain. Whole genome sequencing identified nine BGCs, three of which were assigned to known BNC classes: alkylresorcinol, a PKS-deived antimicrobial compound (Kanda et al., 1975; Gadea et al., 2022), an obafluorin-like NRPS-PKS encoding a tRNA synthesis inhibitor (Scott et al., 2019), and a carotenoid. Consistent with these predictions, several secondary metabolites were detected during batch fermentation, including (i) the antibacterial polyketide troposulfenin (Phippen et al., 2019); (ii) aromatic antimicrobial compounds such as roseobacticide and paracentrone (Duan et al., 2020); (iii) the biosurfactant glycolipid rhamnolipid (Chen et al., 2017); and (iv) multiple pigments, including the yellow-orange carotenoids adonirubin, 2-hydroxyastaxanthin, and adonixanthin, as well as tryptanthrin, a yellow-orange alkaloid with reported antibacterial and antifungal activities (Jahng, 2013). Finally, *Cladosporium allicinum* is an environmental filamentous fungus typically associated with terrestrial habitats and airborne spore communities, although it has also been reported from marine sediments (Lee et al., 2023). Genome mining and fermentation metabolite profiling revealed an extensive biosynthetic repertoire in this isolate, with the detection of 26 BGCs encoding pigments and a wide range of antimicrobial compounds, supporting its role as a naturally optimized microbial cell factory capable of producing multiple classes of high-value secondary metabolites. Consistent with these predictions, several secondary metabolites were detected, including (i) multiple antimicrobial PKSs, such as isocladosporin, cladosporin, cladospolide, cladosporol, cladodionen, and cladosporiumin (Wang et al., 2020; Salvatore et al., 2021; Li H. et al., 2024); (ii) the orange-red pigmented anthraquinone endocrocin, whose ecological role remains incompletely understood but whose related compounds often contribute to UV protection (Raffa and Keller, 2019); and (iii) 1,8-dihydroxynaphthalene, a polyketide-derived precursor of melanin associated with brown to black pigmentation, involved in the protection against UV radiation and oxidative stress (Singh et al., 2021). Taken together, the characterization of isolates obtained from host-associated marine microbiomes highlight the ecological importance of microbial symbionts as protective partner within marine holobionts (Kanda et al., 1975; Duan et al., 2020; Wang et al., 2020; Salvatore et al., 2021; Singh et al., 2021; Gadea et al., 2022; Bailly, 2025). These microorganisms can provide hosts with a diverse arsenal of bioactive metabolites, including antimicrobial compounds for defense against pathogens and protective pigments that shield against UV radiation and oxidative stress. From a biotechnological perspective, this diversity represents a promising source of natural products for the sustainable production of new antimicrobials, preservatives, biomaterials, and natural pigments, the latter for applications in the cosmetic and textile sectors. In particular, the isolates belonging to the species *P. mancusii* and *Cladosporium* combine the capacity to produce multiple BNCs, a relevant diversity of CAZymes and the ability to metabolize plant-derived polysaccharides, thereby suggesting potential applications in circular and sustainable biotechnological production processes. From an ecological perspective, these catabolic capabilities may facilitate adaptation to marine holobionts, where coral mucus and algal exudates provide abundant carbohydrate-based nutrients that can support host colonization and persistence.

## 5 Conclusion

The integration of genomic and metabolomic approaches provides complementary information for the identification and characterization of microorganisms with biotechnological potential. While discrepancies between predicted and detected secondary metabolites may occur due to the complexity of BGC annotation and functional assignment, the combination of these approaches links biosynthetic potential to its actual *in vitro* expression, offering a more reliable assessment of the microorganisms’ biotechnological value. In our work, the combined genomic and metabolomic approach enabled the identification of diverse microorganisms from natural microbiomes with significant biotechnological potential. These strains represent promising microbial cell factories, combining biosynthetic potential for novel BNCs, robust biosynthetic capabilities in laboratory condition, rapid growth, stable biomass accumulation in liquid media, and the production of industrially relevant pigments and bioactive compounds. Among them, a *C. allicinium* strain isolated from the coral *C. inornata* emerged as a particularly promising candidate due to its extensive biosynthetic repertoire, including numerous antimicrobial compounds and pigmented anthraquinones. Similarly, a *P. marcusii* strain associated with the seagrass *P. oceanica* demonstrated the ability to produce multiple carotenoids from plant-derived substrates, highlighting its potential for sustainable pigment production. In addition, two putative novel genomic lineages affiliated with *A. algicola* and *C. oceanosediminis*, isolated from volcanic coastal marine sediments, exhibited strong potential for exploitation in circular bioprocesses aimed at the valorization of agro-industrial side streams into antimicrobials, biosurfactants, and pigmented anthraquinones and carotenoids. Such bio-based outputs could address multiple industrial demands, including pharmaceuticals, feed and food additives, cosmetics, and sustainable textile dyeing. Overall, these findings demonstrate how omics-driven discovery—supported by batch fermentation and scale-aware analytical workflows—can unlock new biotechnological potential of underexplored microbiomes, enabling the identification of metabolically versatile microorganisms capable of supporting circular bioprocessing strategies. By linking microbial diversity with functional biosynthetic capacity, this approach highlights natural microbiomes as valuable reservoirs of next-generation microbial cell factories supporting sustainable and circular bioprocessing strategies.

## Data Availability

All sequencing data are openly available in European Nucleotide Archive (ENA), reference number PRJEB112291.
