# Supplementary material for: Isolation and characterization of novel microorganisms producing natural compounds of possible industrial interest: an integrated genomic and metabolomic approach
Source: Front Microbiol. 2026 Jul 8;17:1872113. doi: 10.3389/fmicb.2026.1872113 (PMC13388484; doi:10.3389/fmicb.2026.1872113)
Supplement: Supplementary file 1 [file Data_Sheet_1.pdf]

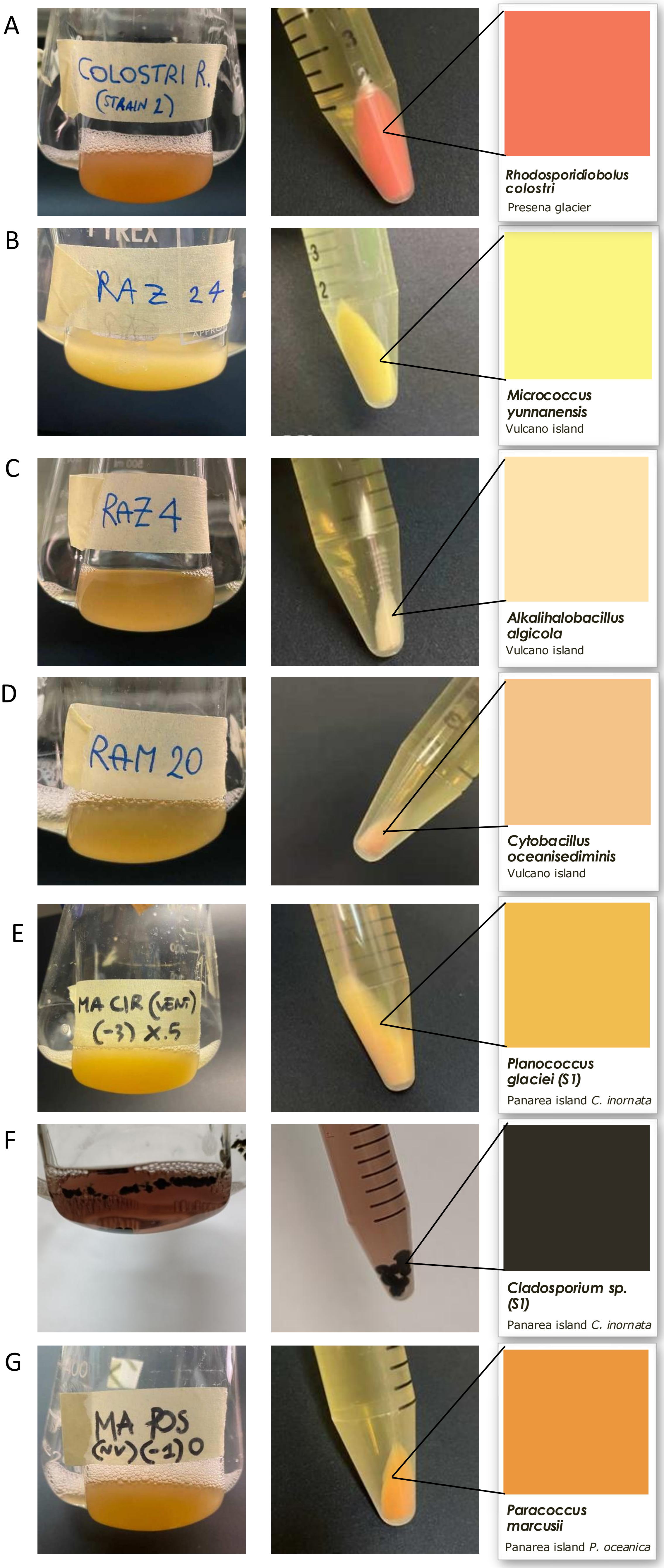

Supplementary Figure 1 – Photographic report of pigmented cell cultures and pellets of selected microorganisms. From the top row: A) *Rhodosporidiobolus colostri*; B) *Micrococcus yunnanensis*; C) *Alkalihalobacillus algicola*; D) *Cytobacillus oceanisediminis*; E) *Planococcus glacei*; F) *Cladosporium sp.*; F) *Paracoccus marcusii*. For *Cladosporium sp.* background color was white to better contrast the darker cell floccules.
