# Supplementary material for: Isolation and characterization of novel microorganisms producing natural compounds of possible industrial interest: an integrated genomic and metabolomic approach
Source: Front Microbiol. 2026 Jul 8;17:1872113. doi: 10.3389/fmicb.2026.1872113 (PMC13388484; doi:10.3389/fmicb.2026.1872113)

Supplementary Figure 3 (3 pages) - Phylogeny of the selected strains reconstructed using ANI distances against NCBI RefSeq/GenBank reference genomes. The isolated strain is shown at the top-left of the corresponding dendogram. The reference strain and its closest reference genome are indicated with asterisks in each dendogram.

Paracoccus marcusii

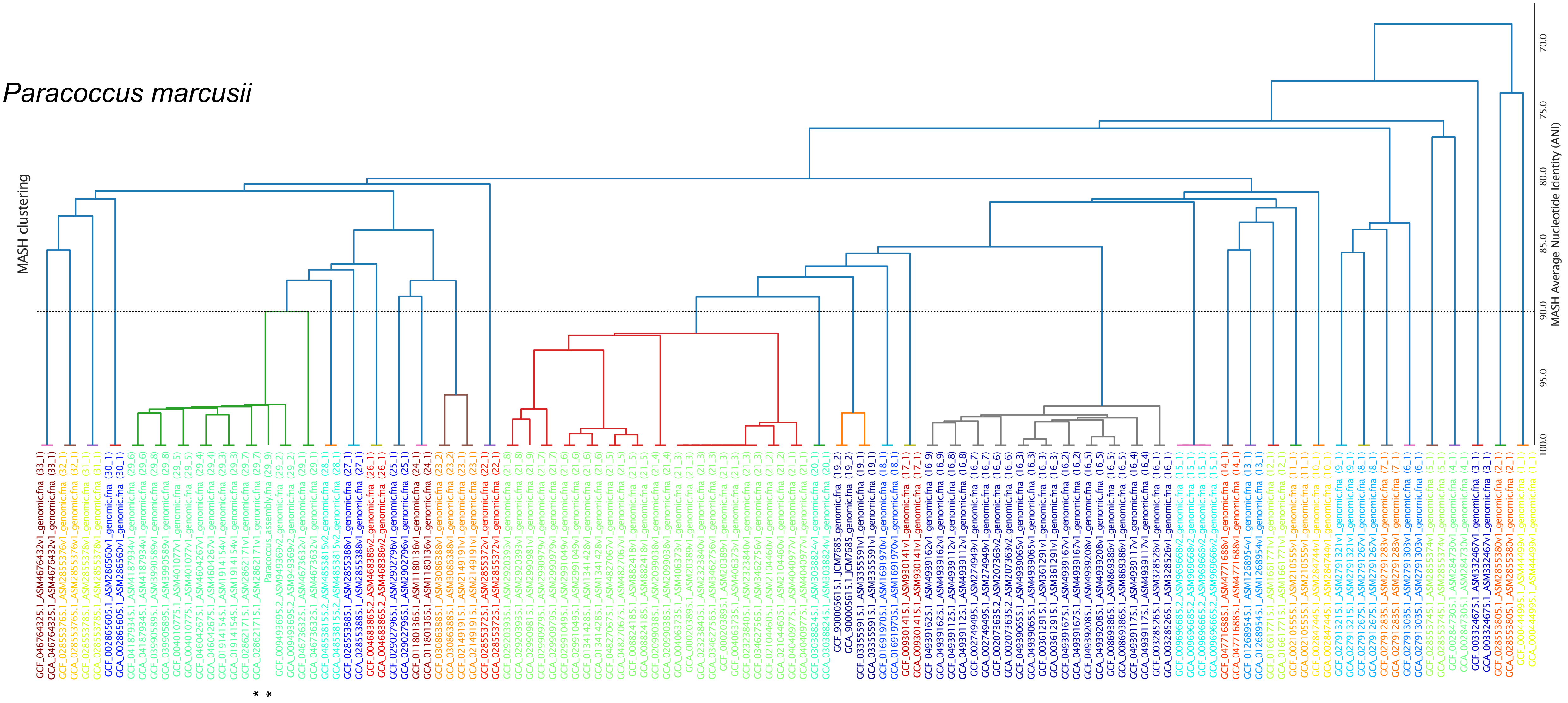

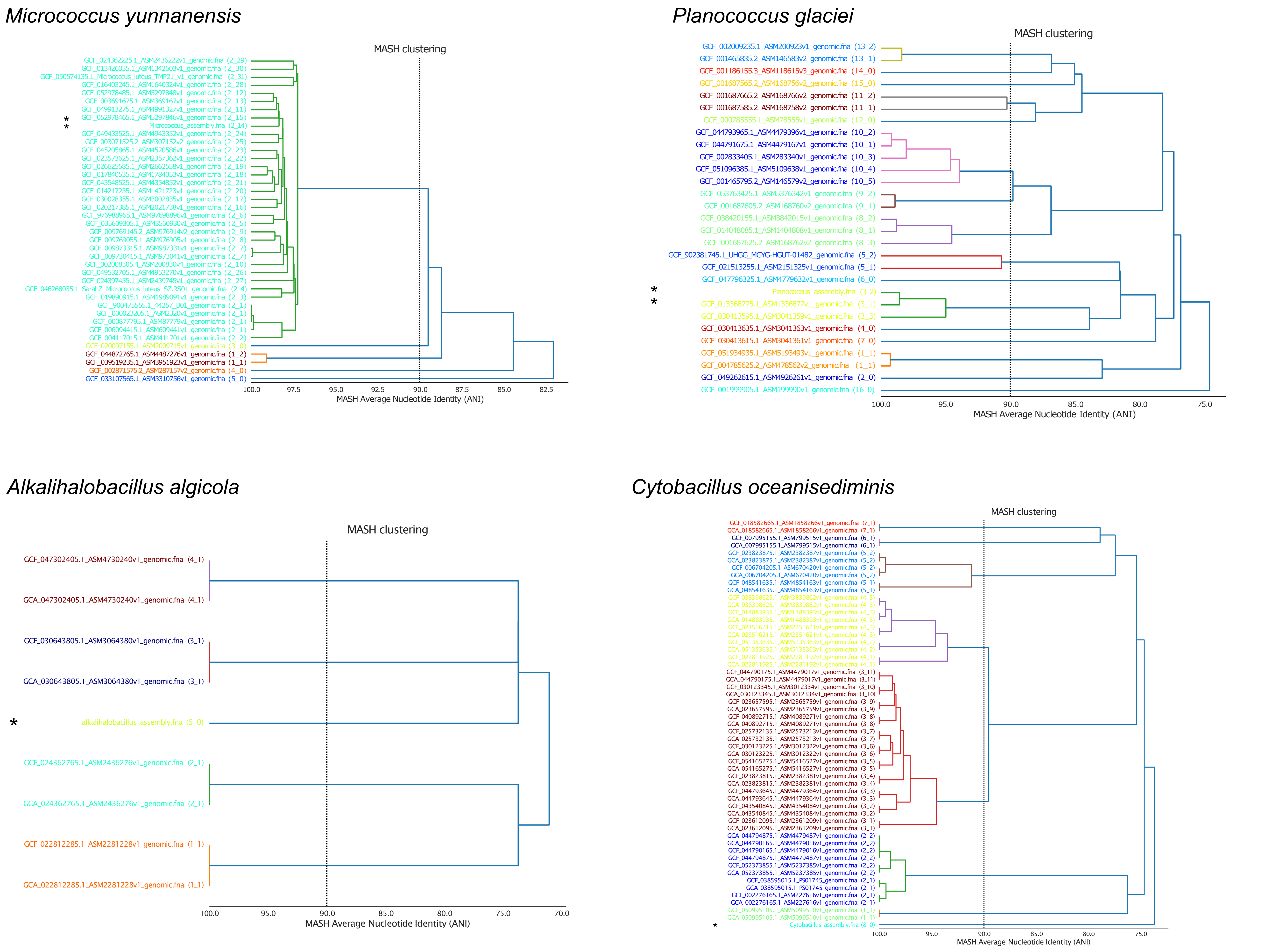

Micrococcus yunnanensis
MASH clustering
GCF_024362225.1_ASM2436222v1_genomic.fna (2_29)
GCF_013426035.1_ASM1342603v1_genomic.fna (2_30)
GCF_050574135.1_Micrococcus_luteus_TMP21_v1_genomic.fna (2_31)
GCF_016403245.1_ASM1640324v1_genomic.fna (2_28)
GCF_052978485.1_ASM5297848v1_genomic.fna (2_12)
GCF_003691675.1_ASM369167v1_genomic.fna (2_13)
GCF_049913275.1_ASM4991327v1_genomic.fna (2_11)
* GCF_052978465.1_ASM5297846v1_genomic.fna (2_15)
* Micrococcus_assembly.fna (2_14)
GCF_049433525.1_ASM4943352v1_genomic.fna (2_24)
GCF_003071525.2_ASM307152v2_genomic.fna (2_25)
GCF_045205865.1_ASM4520586v1_genomic.fna (2_23)
GCF_023573625.1_ASM2357362v1_genomic.fna (2_22)
GCF_026625585.1_ASM2662558v1_genomic.fna (2_19)
GCF_017840535.1_ASM1784053v1_genomic.fna (2_18)
GCF_043548525.1_ASM4354852v1_genomic.fna (2_21)
GCF_014217235.1_ASM1421723v1_genomic.fna (2_20)
GCF_030028355.1_ASM3002835v1_genomic.fna (2_17)
GCF_020217385.1_ASM2021738v1_genomic.fna (2_16)
GCF_976988965.1_ASM97698896v1_genomic.fna (2_6)
GCF_035609305.1_ASM3560930v1_genomic.fna (2_5)
GCF_009769145.2_ASM976914v2_genomic.fna (2_9)
GCF_009769055.1_ASM976905v1_genomic.fna (2_8)
GCF_009873315.1_ASM987331v1_genomic.fna (2_7)
GCF_009730415.1_ASM973041v1_genomic.fna (2_7)
GCF_002008305.4_ASM200830v4_genomic.fna (2_10)
GCF_049532705.1_ASM4953270v1_genomic.fna (2_26)
GCF_024397455.1_ASM2439745v1_genomic.fna (2_27)
GCF_046268035.1_SarahZ_Micrococcus_luteus_SZ.RS01_genomic.fna (2_4)
GCF_019890915.1_ASM1989091v1_genomic.fna (2_3)
GCF_900475555.1_44257_B01_genomic.fna (2_1)
GCF_000023205.1_ASM2320v1_genomic.fna (2_1)
GCF_000877795.1_ASM87779v1_genomic.fna (2_1)
GCF_006094415.1_ASM609441v1_genomic.fna (2_1)
GCF_004117015.1_ASM411701v1_genomic.fna (2_2)
GCF_020097155.1_ASM2009715v1_genomic.fna (3_0)
GCF_044872765.1_ASM4487276v1_genomic.fna (1_2)
GCF_039519235.1_ASM3951923v1_genomic.fna (1_1)
GCF_002871575.2_ASM287157v2_genomic.fna (4_0)
GCF_033107565.1_ASM3310756v1_genomic.fna (5_0)
100.0
97.5
95.0
92.5
90.0
87.5
85.0
82.5
MASH Average Nucleotide Identity (ANI)
Planococcus glaciei
MASH clustering
GCF_002009235.1_ASM200923v1_genomic.fna (13_2)
GCF_001465835.2_ASM146583v2_genomic.fna (13_1)
GCF_001186155.3_ASM118615v3_genomic.fna (14_0)
GCF_001687565.2_ASM168756v2_genomic.fna (15_0)
GCF_001687665.2_ASM168766v2_genomic.fna (11_2)
GCF_001687585.2_ASM168758v2_genomic.fna (11_1)
GCF_000785555.1_ASM78555v1_genomic.fna (12_0)
GCF_044793965.1_ASM4479396v1_genomic.fna (10_2)
GCF_044791675.1_ASM4479167v1_genomic.fna (10_1)
GCF_002833405.1_ASM283340v1_genomic.fna (10_3)
GCF_051096385.1_ASM5109638v1_genomic.fna (10_4)
GCF_001465795.2_ASM146579v2_genomic.fna (10_5)
GCF_053763425.1_ASM5376342v1_genomic.fna (9_2)
GCF_001687605.2_ASM168760v2_genomic.fna (9_1)
GCF_038420155.1_ASM3842015v1_genomic.fna (8_2)
GCF_014048085.1_ASM1404808v1_genomic.fna (8_1)
GCF_001687625.2_ASM168762v2_genomic.fna (8_3)
GCF_902381745.1_UHGG_MGYG-HGUT-01482_genomic.fna (5_2)
GCF_021513255.1_ASM2151325v1_genomic.fna (5_1)
GCF_047796325.1_ASM4779632v1_genomic.fna (6_0)
* Planococcus_assembly.fna (3_2)
* GCF_013368775.1_ASM1336877v1_genomic.fna (3_1)
GCF_030413595.1_ASM3041359v1_genomic.fna (3_3)
GCF_030413635.1_ASM3041363v1_genomic.fna (4_0)
GCF_030413615.1_ASM3041361v1_genomic.fna (7_0)
GCF_051934935.1_ASM5193493v1_genomic.fna (1_1)
GCF_004785625.2_ASM478562v2_genomic.fna (1_1)
GCF_049262615.1_ASM4926261v1_genomic.fna (2_0)
GCF_001999905.1_ASM199990v1_genomic.fna (16_0)
100.0
95.0
90.0
85.0
80.0
75.0
MASH Average Nucleotide Identity (ANI)
Alkalihalobacillus algicola
MASH clustering
GCF_047302405.1_ASM4730240v1_genomic.fna (4_1)
GCA_047302405.1_ASM4730240v1_genomic.fna (4_1)
GCF_030643805.1_ASM3064380v1_genomic.fna (3_1)
GCA_030643805.1_ASM3064380v1_genomic.fna (3_1)
* alkalihalobacillus_assembly.fna (5_0)
GCF_024362765.1_ASM2436276v1_genomic.fna (2_1)
GCA_024362765.1_ASM2436276v1_genomic.fna (2_1)
GCF_022812285.1_ASM2281228v1_genomic.fna (1_1)
GCA_022812285.1_ASM2281228v1_genomic.fna (1_1)
100.0
95.0
90.0
85.0
80.0
75.0
70.0
MASH Average Nucleotide Identity (ANI)
Cytobacillus oceanisediminis
MASH clustering
GCF_018582665.1_ASM1858266v1_genomic.fna (7_1)
GCA_018582665.1_ASM1858266v1_genomic.fna (7_1)
GCF_007995155.1_ASM799515v1_genomic.fna (6_1)
GCA_007995155.1_ASM799515v1_genomic.fna (6_1)
GCF_023823875.1_ASM2382387v1_genomic.fna (5_2)
GCA_023823875.1_ASM2382387v1_genomic.fna (5_2)
GCF_006704205.1_ASM670420v1_genomic.fna (5_2)
GCA_006704205.1_ASM670420v1_genomic.fna (5_2)
GCF_048541635.1_ASM4854163v1_genomic.fna (5_1)
GCA_048541635.1_ASM4854163v1_genomic.fna (5_1)
GCF_038398625.1_ASM3839862v1_genomic.fna (4_3)
GCA_038398625.1_ASM3839862v1_genomic.fna (4_3)
GCF_014883935.1_ASM1488393v1_genomic.fna (4_3)
GCA_014883935.1_ASM1488393v1_genomic.fna (4_3)
GCF_023516215.1_ASM2351621v1_genomic.fna (4_3)
GCA_023516215.1_ASM2351621v1_genomic.fna (4_3)
GCF_051353635.1_ASM5135363v1_genomic.fna (4_2)
GCA_051353635.1_ASM5135363v1_genomic.fna (4_2)
GCF_022811925.1_ASM2281192v1_genomic.fna (4_1)
GCA_022811925.1_ASM2281192v1_genomic.fna (4_1)
GCF_044790175.1_ASM4479017v1_genomic.fna (3_11)
GCA_044790175.1_ASM4479017v1_genomic.fna (3_11)
GCF_030123345.1_ASM3012334v1_genomic.fna (3_10)
GCA_030123345.1_ASM3012334v1_genomic.fna (3_10)
GCF_023657595.1_ASM2365759v1_genomic.fna (3_9)
GCA_023657595.1_ASM2365759v1_genomic.fna (3_9)
GCF_040892715.1_ASM4089271v1_genomic.fna (3_8)
GCA_040892715.1_ASM4089271v1_genomic.fna (3_8)
GCF_025732135.1_ASM2573213v1_genomic.fna (3_7)
GCA_025732135.1_ASM2573213v1_genomic.fna (3_7)
GCF_030123225.1_ASM3012322v1_genomic.fna (3_6)
GCA_030123225.1_ASM3012322v1_genomic.fna (3_6)
GCF_054165275.1_ASM5416527v1_genomic.fna (3_5)
GCA_054165275.1_ASM5416527v1_genomic.fna (3_5)
GCF_023823815.1_ASM2382381v1_genomic.fna (3_4)
GCA_023823815.1_ASM2382381v1_genomic.fna (3_4)
GCF_044793645.1_ASM4479364v1_genomic.fna (3_3)
GCA_044793645.1_ASM4479364v1_genomic.fna (3_3)
GCF_043540845.1_ASM4354084v1_genomic.fna (3_2)
GCA_043540845.1_ASM4354084v1_genomic.fna (3_2)
GCF_023612095.1_ASM2361209v1_genomic.fna (3_1)
GCA_023612095.1_ASM2361209v1_genomic.fna (3_1)
GCA_044794875.1_ASM4479487v1_genomic.fna (2_2)
GCA_044790165.1_ASM4479016v1_genomic.fna (2_2)
GCF_044790165.1_ASM4479016v1_genomic.fna (2_2)
GCF_044794875.1_ASM4479487v1_genomic.fna (2_2)
GCF_052373855.1_ASM5237385v1_genomic.fna (2_2)
GCA_052373855.1_ASM5237385v1_genomic.fna (2_2)
GCF_038595015.1_PS01745_genomic.fna (2_1)
GCA_038595015.1_PS01745_genomic.fna (2_1)
GCF_002276165.1_ASM227616v1_genomic.fna (2_1)
GCA_002276165.1_ASM227616v1_genomic.fna (2_1)
GCF_050995105.1_ASM5099510v1_genomic.fna (1_1)
GCA_050995105.1_ASM5099510v1_genomic.fna (1_1)
* Cytobacillus_assembly.fna (8_0)
100.0
95.0
90.0
85.0
80.0
75.0
MASH Average Nucleotide Identity (ANI)

Rhodosporidiobolus colostri

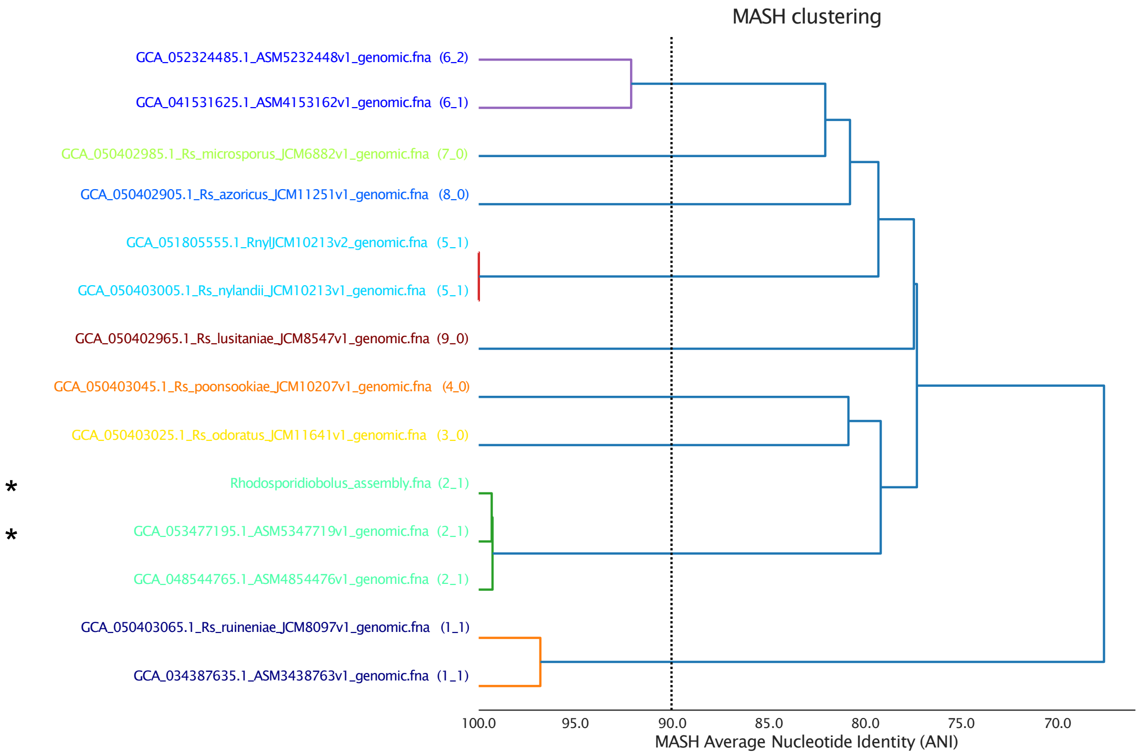

Cladosporium sp.

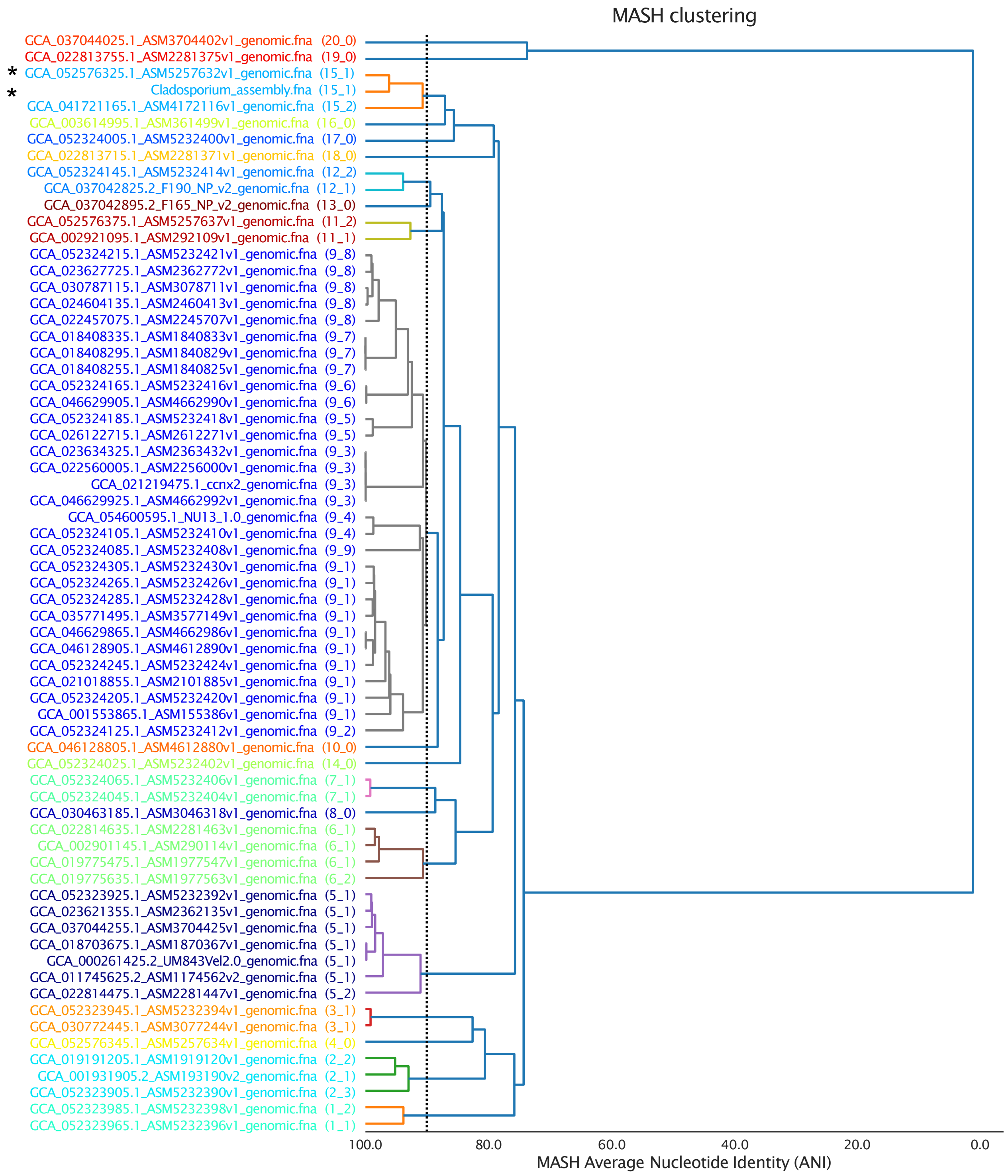

Supplement: Supplementary file 3 [file Data_Sheet_3.pdf]
