# Supplementary material for: Isolation and characterization of novel microorganisms producing natural compounds of possible industrial interest: an integrated genomic and metabolomic approach
Source: Front Microbiol. 2026 Jul 8;17:1872113. doi: 10.3389/fmicb.2026.1872113 (PMC13388484; doi:10.3389/fmicb.2026.1872113)

Supplementary Figure 4 (6 pages) – Pangenomes construction and comparative genomics. For *Cytobacillus oceanisediminis* (A) and *Micrococcus yunnanensis* (B), an in-depth comparative genomic assessment was performed. Genome assemblies generated in this study, together with publicly available genomes retrieved from GenBank, were structurally and functionally annotated to reconstruct species-specific pangenomes. Genomic similarity was evaluated using the Jaccard distance calculated from the gene presence/absence matrix derived from each pangenome. Relationships among genomes were then investigated through Principal Coordinates Analysis (PCoA) based on the Jaccard distance matrix (1), as well as by constructing Neighbor-Joining (NJ) (2) and UPGMA clustering trees (3). These analyses enabled the assessment of the genomic diversity within each species and the degree of novelty represented by the newly isolated strains.

A (1)

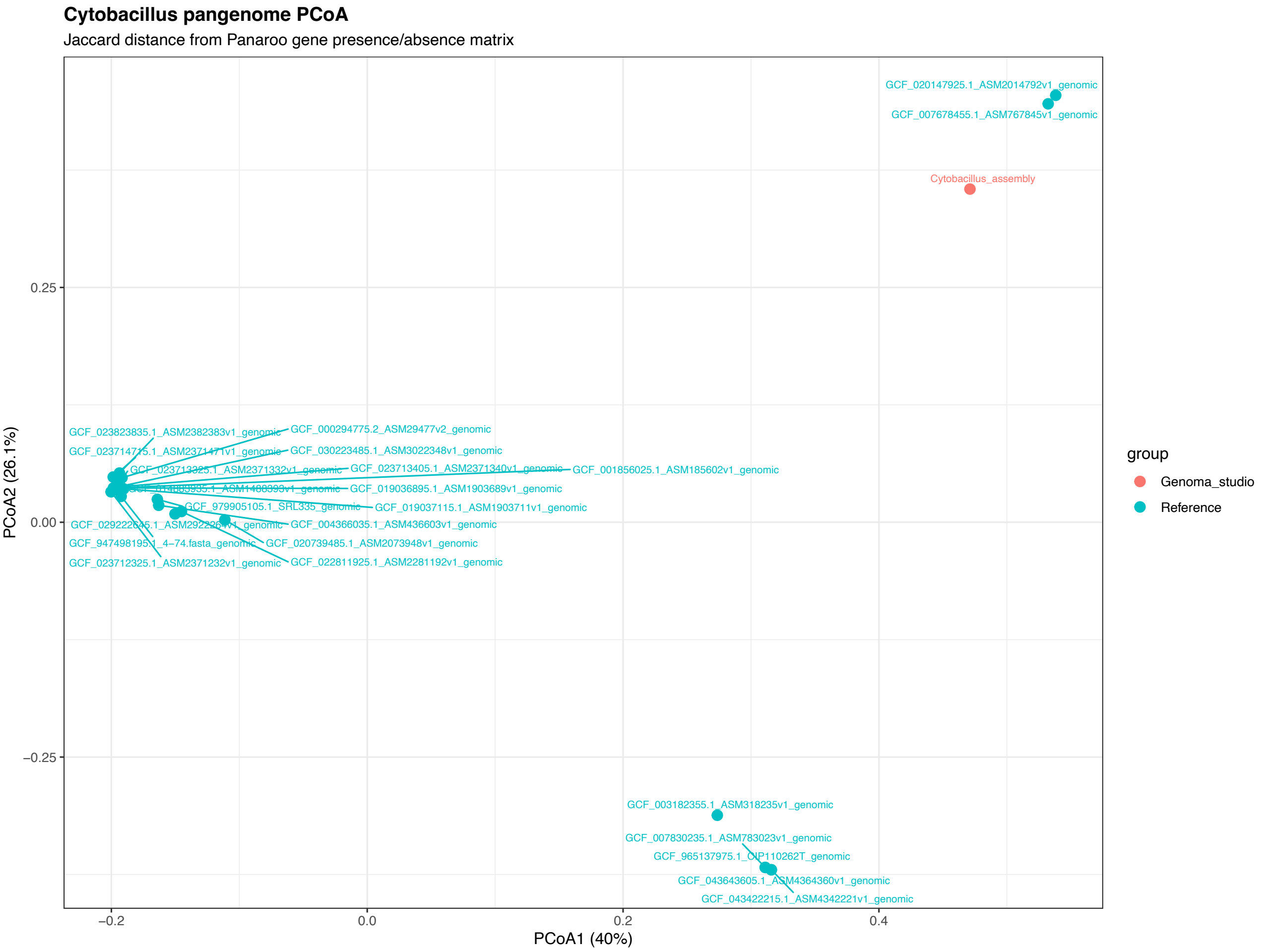

A (2)

Cytobacillus Panaroo/Jaccard Neighbor–Joining tree

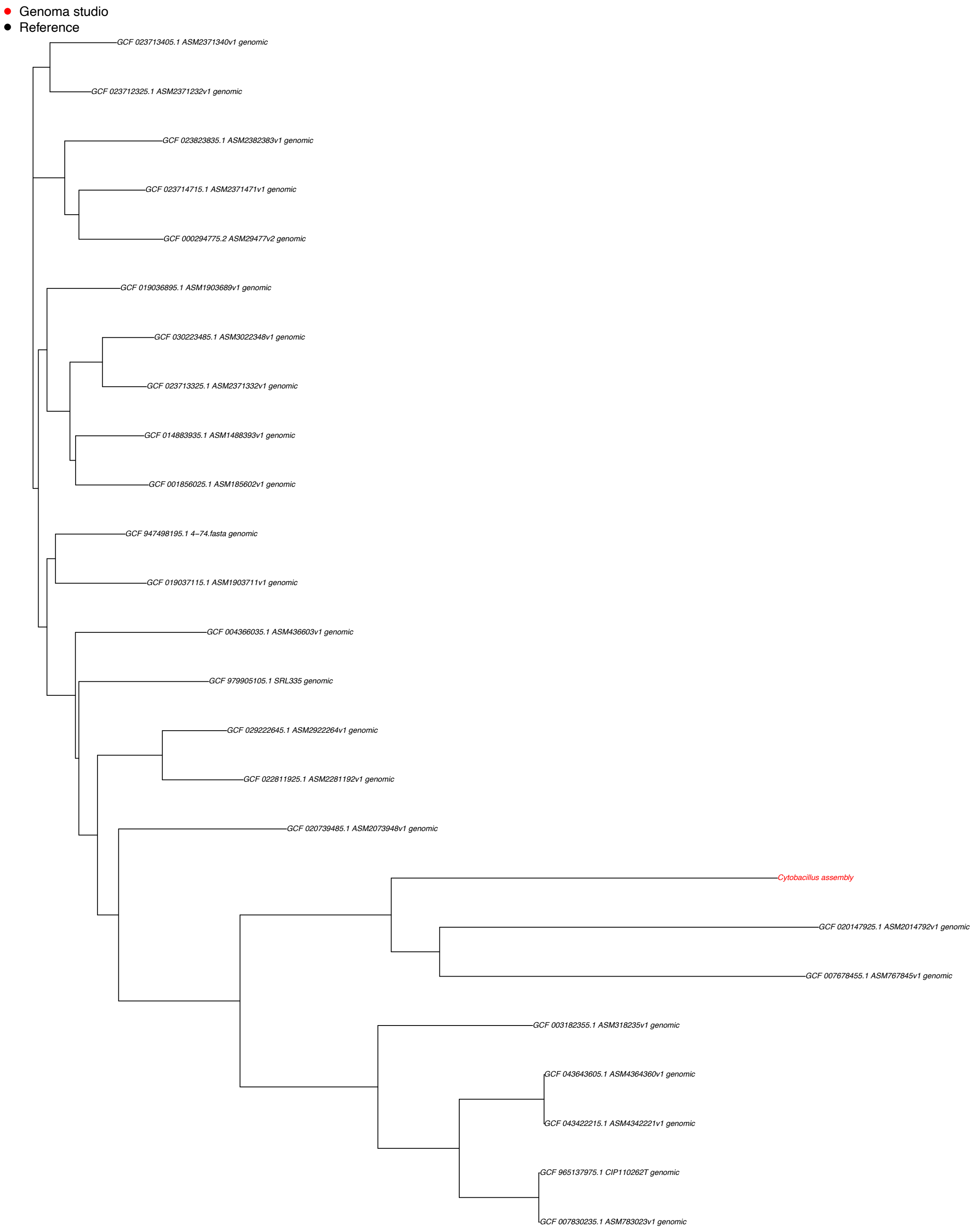

A (3)

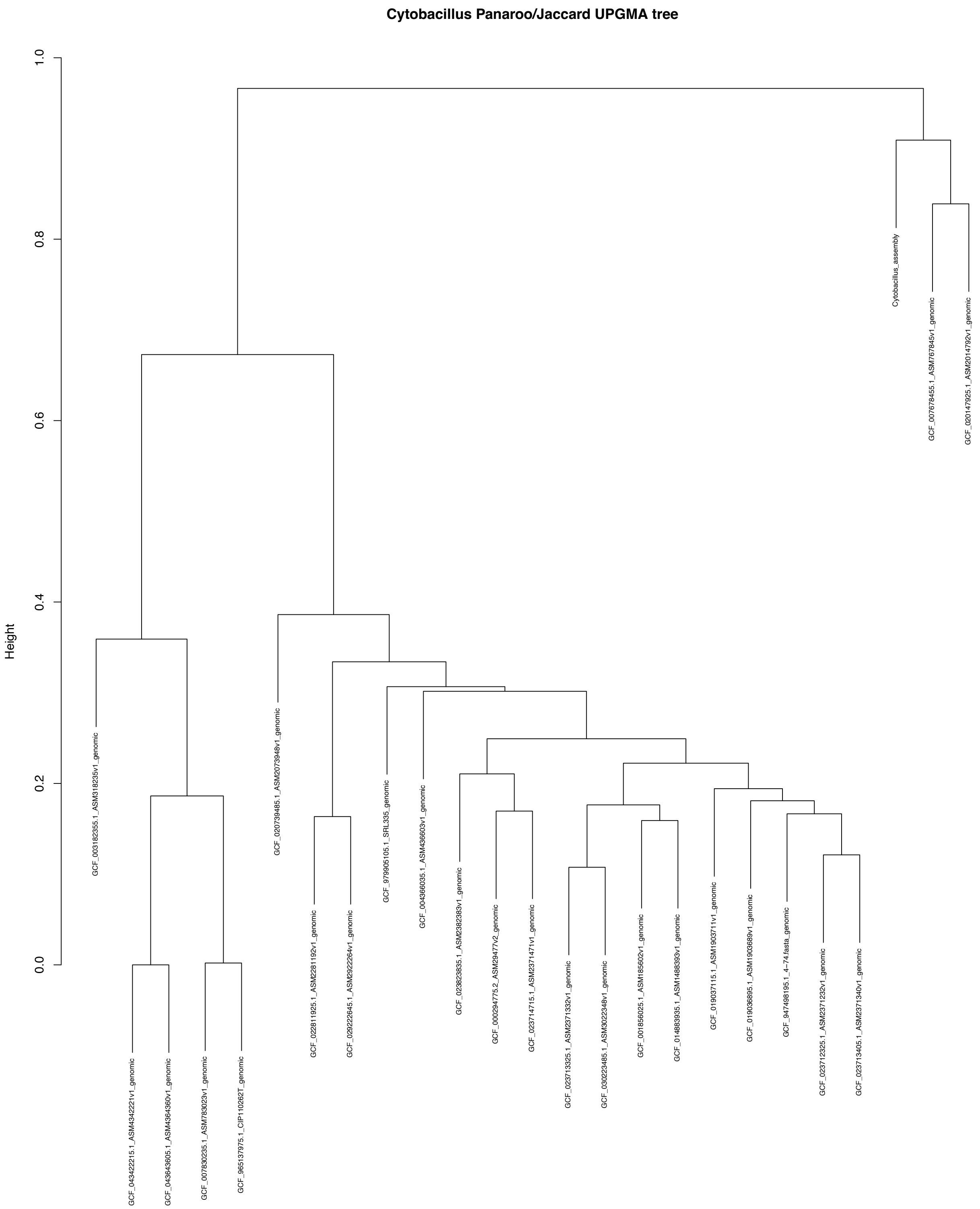
Cytobacillus Panaroo/Jaccard UPGMA tree
Height
1.0
0.8
0.6
0.4
0.2
0.0
GCF_003182355.1_ASM318235v1_genomic
GCF_043422215.1_ASM4342221v1_genomic
GCF_043643605.1_ASM4364360v1_genomic
GCF_007830235.1_ASM783023v1_genomic
GCF_965137975.1_CIP110262T_genomic
GCF_020739485.1_ASM2073948v1_genomic
GCF_022811925.1_ASM2281192v1_genomic
GCF_029222645.1_ASM2922264v1_genomic
GCF_979905105.1_SRL335_genomic
GCF_004366035.1_ASM436603v1_genomic
GCF_023823835.1_ASM2382383v1_genomic
GCF_000294775.2_ASM29477v2_genomic
GCF_023714715.1_ASM2371471v1_genomic
GCF_023713325.1_ASM2371332v1_genomic
GCF_030223485.1_ASM3022348v1_genomic
GCF_001856025.1_ASM185602v1_genomic
GCF_014883935.1_ASM1488393v1_genomic
GCF_019037115.1_ASM1903711v1_genomic
GCF_019036895.1_ASM1903689v1_genomic
GCF_947498195.1_4−74.fasta_genomic
GCF_023712325.1_ASM2371232v1_genomic
GCF_023713405.1_ASM2371340v1_genomic
Cytobacillus_assembly
GCF_007678455.1_ASM767845v1_genomic
GCF_020147925.1_ASM2014792v1_genomic

B (1)

Micrococcus pangenome PCoA

Jaccard distance from Panaroo gene presence/absence matrix

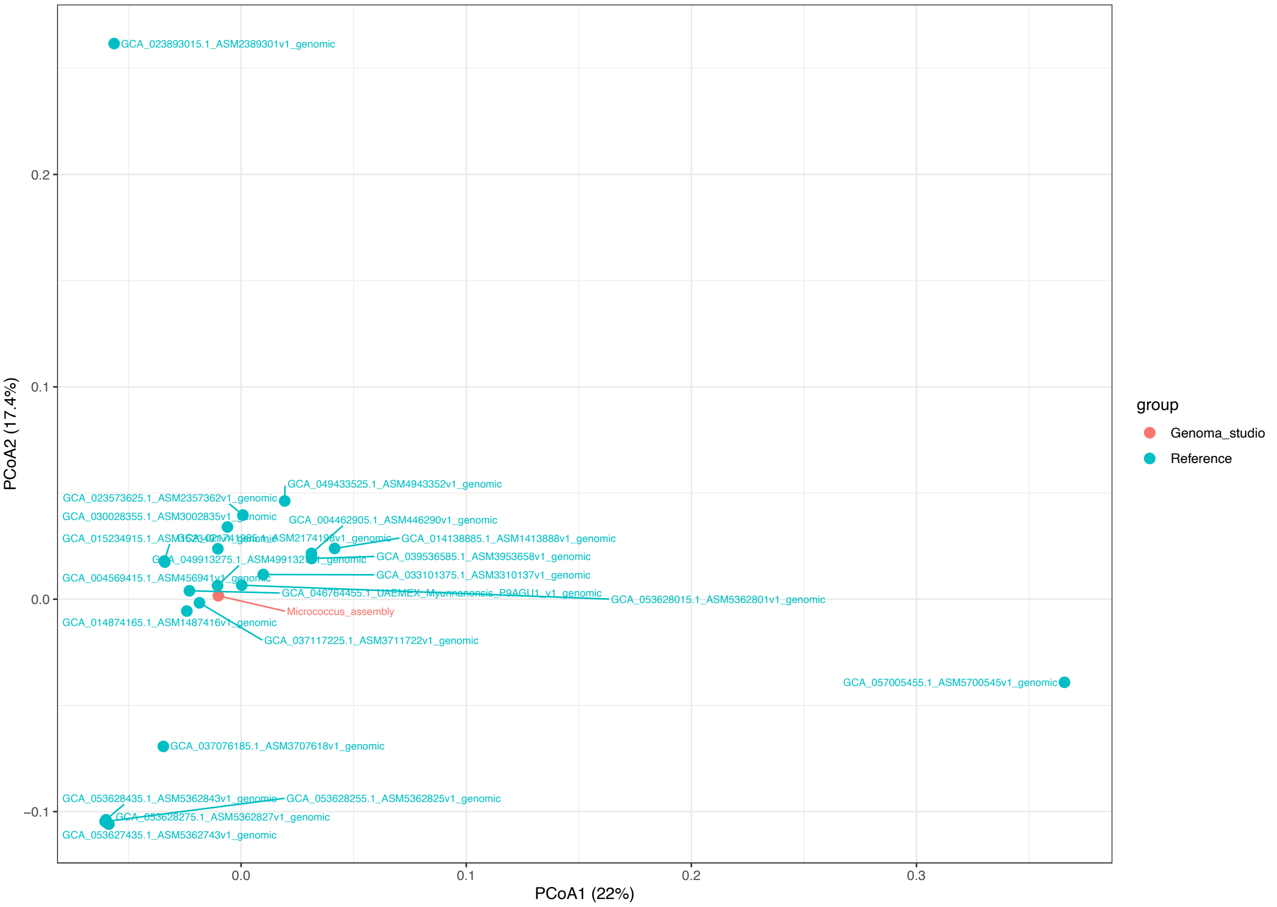

B (2)

Micrococcus Panaroo/Jaccard Neighbor–Joining tree

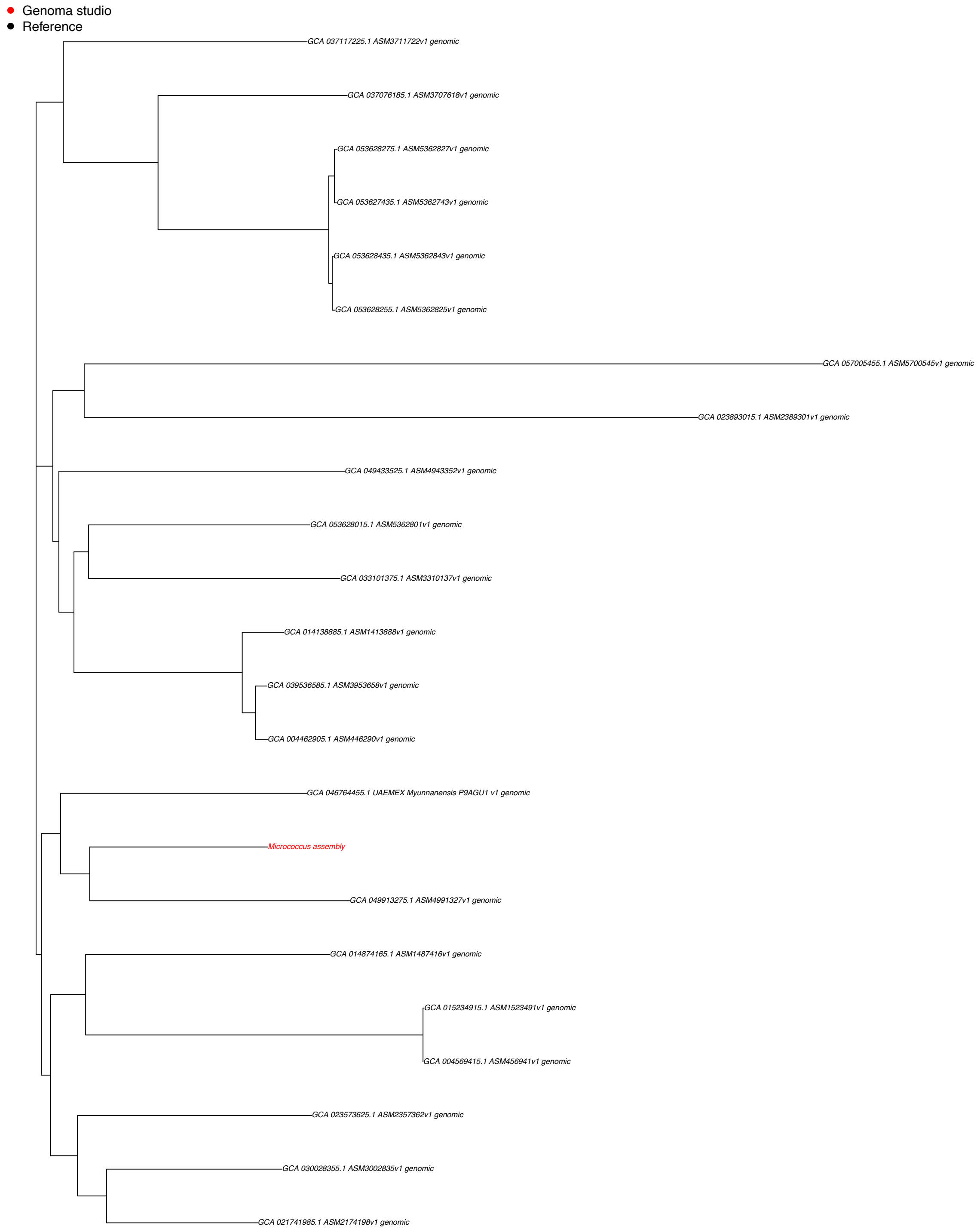

B (3)

Micrococcus Panaroo/Jaccard UPGMA tree

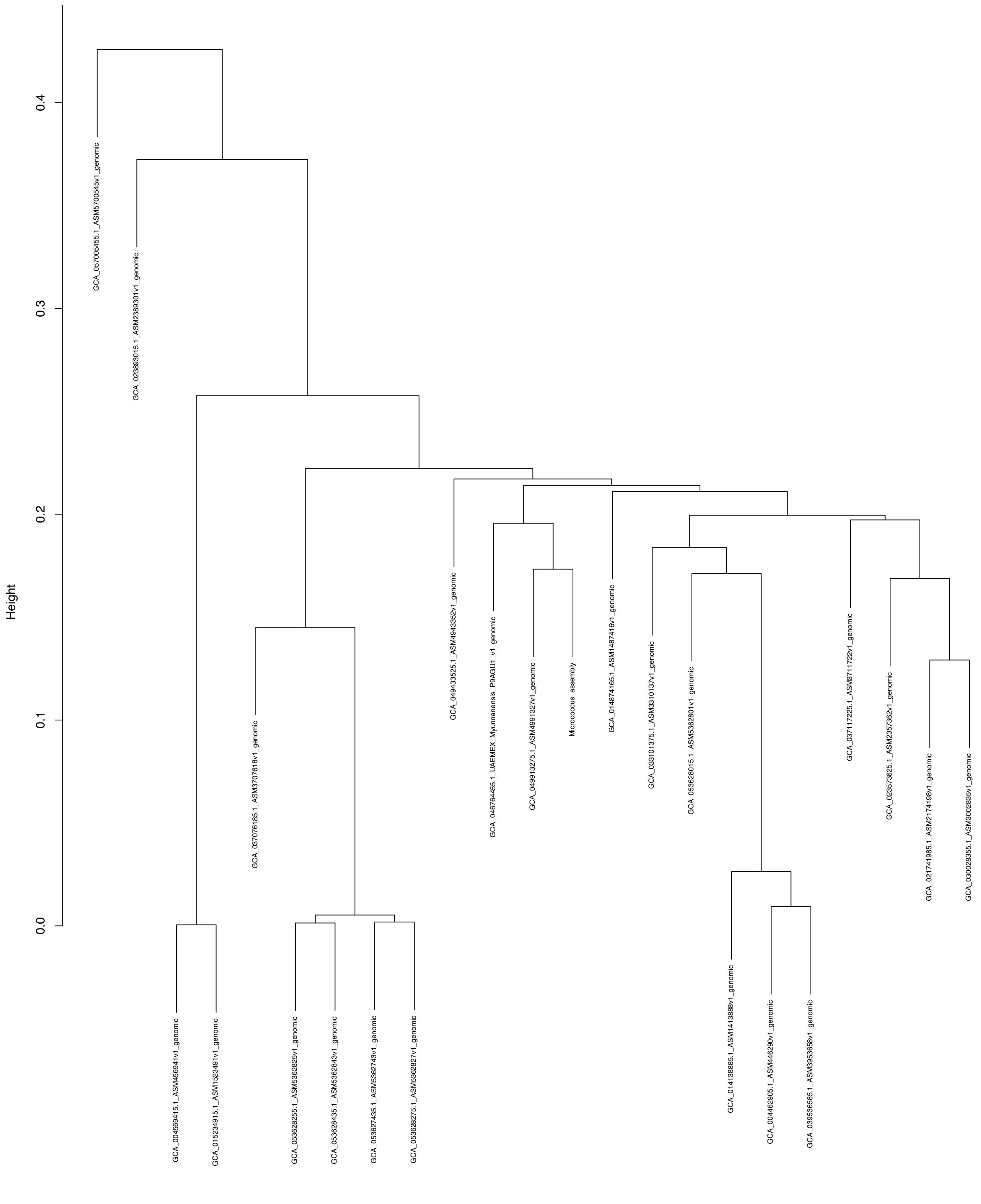

Supplement: Supplementary file 4 [file Data_Sheet_4.pdf]
