# Supplementary material for: Isolation and characterization of novel microorganisms producing natural compounds of possible industrial interest: an integrated genomic and metabolomic approach
Source: Front Microbiol. 2026 Jul 8;17:1872113. doi: 10.3389/fmicb.2026.1872113 (PMC13388484; doi:10.3389/fmicb.2026.1872113)
Supplement: Supplementary file 2 [file Data_Sheet_2.pdf]

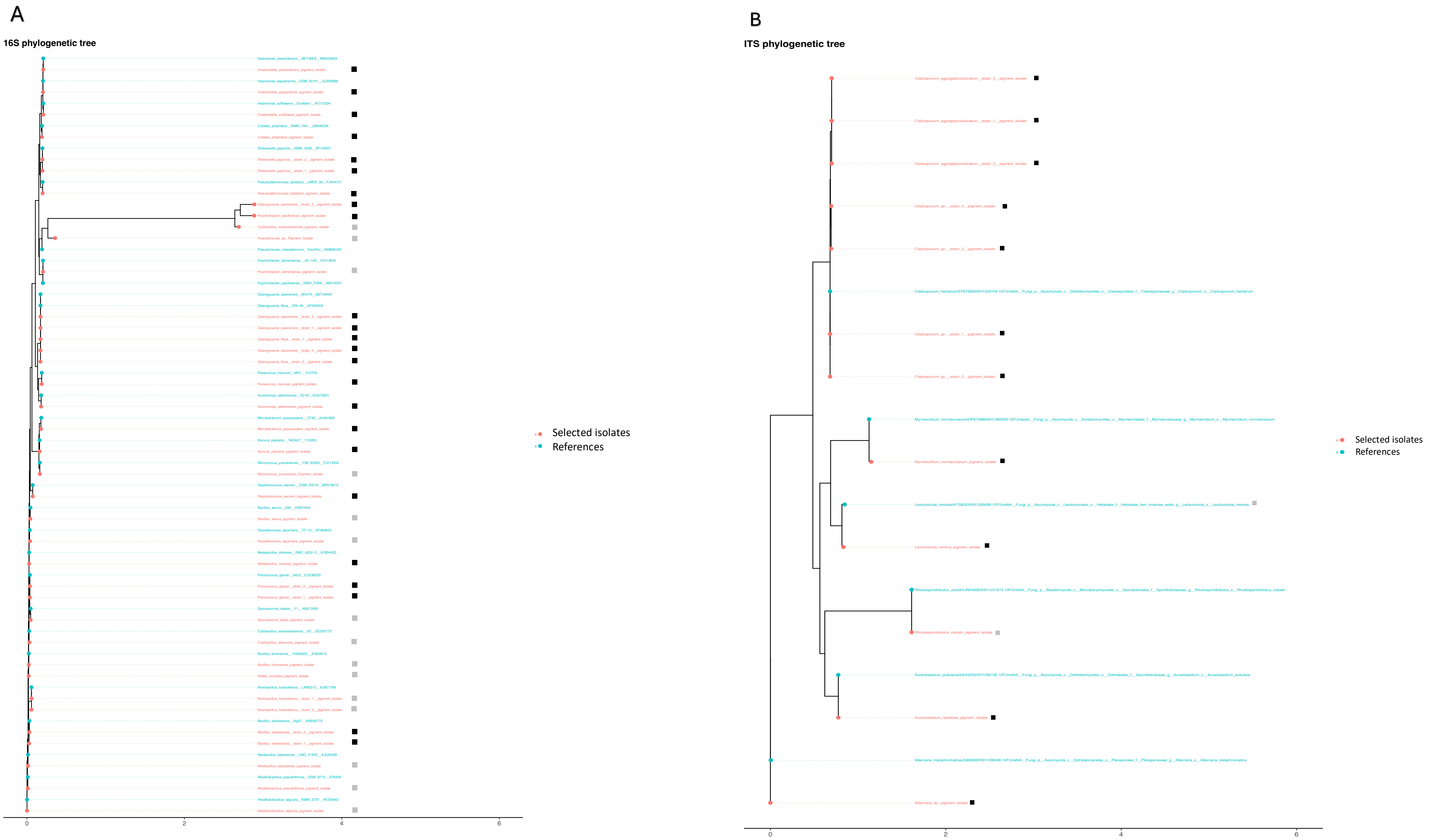


**Supplementary Figure 2 – Phylogenetic analyses based on 16S rRNA gene and ITS sequences.** A) Phylogenetic relationships of bacterial pigment-producing isolates inferred from 16S rRNA gene sequences. Study isolates were clustered together with their closest reference sequences retrieved from public databases. The tree was constructed from pairwise sequence distances and visualized as a hierarchical clustering dendrogram. B) Phylogenetic relationships of fungal pigment-producing isolates inferred from ITS sequences. Study isolates were compared with reference sequences. Study isolates (red); reference sequences (blue); environmental (grey); host-associated (black).
