# Supplementary material for: Isolation and characterization of novel microorganisms producing natural compounds of possible industrial interest: an integrated genomic and metabolomic approach
Source: Front Microbiol. 2026 Jul 8;17:1872113. doi: 10.3389/fmicb.2026.1872113 (PMC13388484; doi:10.3389/fmicb.2026.1872113)

Supplementary Figure 5 - CAZyme family profile for the isolated strains. Carbohydrate-active enzymes (CAZymes) were annotated for each isolate to provide a a comprehensive glycobiome profile for all taxa.

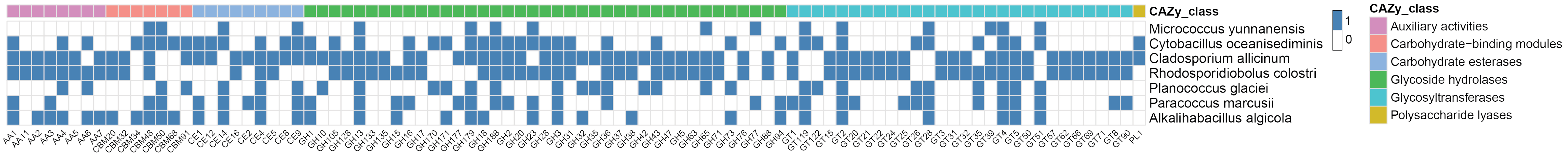

Supplement: Supplementary file 5 [file Data_Sheet_5.pdf]
